# Understanding the Distribution of Marine Megafauna in the English Channel Region: Identifying Key Habitats for Conservation within the Busiest Seaway on Earth

**DOI:** 10.1371/journal.pone.0089720

**Published:** 2014-02-28

**Authors:** Catherine M. McClellan, Tom Brereton, Florence Dell'Amico, David G. Johns, Anna-C. Cucknell, Samantha C. Patrick, Rod Penrose, Vincent Ridoux, Jean-Luc Solandt, Eric Stephan, Stephen C. Votier, Ruth Williams, Brendan J. Godley

**Affiliations:** 1 Centre for Ecology and Conservation, University of Exeter, Penryn, Cornwall, United Kingdom; 2 Marinelife, Bridport, Dorset, United Kingdom; 3 Centre d'Études et de Soins pour les Tortues Marines, Aquarium La Rochelle, La Rochelle, France; 4 Sir Alister Hardy Foundation for Ocean Science, Plymouth, United Kingdom; 5 International Fund for Animal Welfare and Marine Conservation Research International, Kelvedon, Essex, United Kingdom; 6 Biosciences, University of Gloucestershire, Cheltenham, Gloucestershire, United Kingdom; 7 Marine Environmental Monitoring, Llechryd, Ceredigion, United Kingdom; 8 Observatoire PELAGIS, Université de La Rochelle-CNRS, La Rochelle, France; 9 Marine Conservation Society, Ross on Wye, Herefordshire, United Kingdom; 10 Association Pour l'Etude et la Conservation des Sélaciens, Brest, France; 11 Environment and Sustainability Institute, University of Exeter, Penryn, Cornwall, United Kingdom; 12 Cornwall Wildlife Trust, Truro, Cornwall, United Kingdom; National Oceanic and Atmospheric Administration/National Marine Fisheries Service/Southwest Fisheries Science Center, United States of America

## Abstract

The temperate waters of the North-Eastern Atlantic have a long history of maritime resource richness and, as a result, the European Union is endeavouring to maintain regional productivity and biodiversity. At the intersection of these aims lies potential conflict, signalling the need for integrated, cross-border management approaches. This paper focuses on the marine megafauna of the region. This guild of consumers was formerly abundant, but is now depleted and protected under various national and international legislative structures. We present a meta-analysis of available megafauna datasets using presence-only distribution models to characterise suitable habitat and identify spatially-important regions within the English Channel and southern bight of the North Sea. The integration of studies from dedicated and opportunistic observer programmes in the United Kingdom and France provide a valuable perspective on the spatial and seasonal distribution of various taxonomic groups, including large pelagic fishes and sharks, marine mammals, seabirds and marine turtles. The Western English Channel emerged as a hotspot of biodiversity for megafauna, while species richness was low in the Eastern English Channel. Spatial conservation planning is complicated by the highly mobile nature of marine megafauna, however they are important components of the marine environment and understanding their distribution is a first crucial step toward their inclusion into marine ecosystem management.

## Introduction

Awareness and understanding of the consequences of increased anthropogenic pressure in the marine environment is an important global issue. Development of national and international maritime policies such as those recently enacted in the European Union (EU) [1] – a region that relies heavily on marine-based resource exploitation – acknowledges the need for maintaining marine ecosystem integrity. The English Channel, as a case in point, lays claim to the world's busiest seaway and is ranked among the most highly affected marine ecosystem on earth [2]. Here, shipping, fisheries, mariculture, coastal and marine tourism, and submarine mining are just some of the commercial industries operated by multiple nations, which generate great revenue but have the potential to have deleterious environmental impact [3]–[4]. Moreover, as the search for renewable sources of energy advances to meet the needs of growing human populations, anthropogenic pressures are intensifying in this area rather than subsiding. Therefore, there is a pressing need for ecosystem-based management through integrated marine spatial planning across international borders [5]–[6].

Successful management of ocean ecosystems requires adequate knowledge of the species present and their distributions in order to assess realised and potential interactions with anthropogenic activities. Apex predators such as dolphins, whales, sharks, seals, seabirds and marine turtles, together known as marine megafauna, are arguably some of the more iconic members that make up the oceans' biodiversity, yet their distributions, abundance, and functional influence on the ecosystem remains poorly understood. Due to their life history traits (i.e. few offspring, slow growth, late age to maturity) [7], many marine megafauna populations have declined due to unsustainable direct exploitation or incidental mortality [8]–[13]. Several national and international legislative frameworks now attempt to promote the recovery of what populations remain by limiting take and trade of species and/or by restricting human activities in vital habitats (e.g. (IWC) International Convention for the Regulation of Whaling, (Bonn) Convention on Migratory Species, (CITES) Convention in International Trade in Endangered Species of wildlife fauna and flora, (OSPAR) Convention for the Protection of the marine Environment of the North-East Atlantic, (Bern) Convention on the Conservation of European Wildlife and Natural Habitats, European Marine Strategy Framework Directive, European Union Biodiversity Strategy, European Union Habitat Directive, European Birds Directive, (UKBS) United Kingdom Biodiversity Strategy (formerly Biodiversity Action Plan).

Studies that provide the critical information required for a basic ecological understanding of pelagic species are always constrained by tradeoffs imposed by the great cost to access these animals, by obtaining permissions from appropriate authorities, by weather and sea state, by daylight and allotted time to accomplish the study, and by physical risk to either the observer(s) or animal(s). Furthermore, the cryptic and vagile natures of these animals, their sizes, speeds and diving abilities, as well as their long life spans impede a complete scientific understanding. Nevertheless, a wealth of data already exists in the form of observations of marine megafauna from shore-based, at-sea, aerial or animal platforms gathered by both specialists and the wider public, which could be used to help meet the pressing need of decision makers to protect these populations while allowing for resource use at the same time.

The present work explores the spatial conservation planning potential of megafauna data archives by combining existing species occurrence datasets within a focal region into a common meta-analysis that expands the bounds (e.g. season, year, geographic extent, species) of individual data sources. Our aim was to incorporate quantitative methodologies for investigating both individual species' ecologies and multi-species communities that broadly share habitats as a proof of concept that might benefit marine management with results from selected examples. Complications faced during such an undertaking are daunting and not limited to issues of differing data quality, purpose, sampling methods, geographic and temporal scales, seasonality, specificity, effort, duration, and number of records [14]–[16]. However, advances in statistical modelling (namely Bayesian probability theory) provide a way forward for tapping into these valuable yet possibly challenging data. Species distribution models estimate the statistical relationship between species records and their background environment from empirical data at particular sites to predict their distributions into unobserved sites [17]. While quite powerful, traditional habitat modelling methods assume data independence and require absence, pseudo-absence, or abundance data to estimate species' distributions across geographic space [18]–[19] and would not allow incorporation of the vast majority of datasets made available to us. Maximum entropy techniques however, can predict a species' niche using presence-only records and do not require inclusion of sampling effort or assume independence in the data [20]–[21] thus allowing the maximum inclusion of data sources for our study. Maximum entropy modelling has, for that reason, become an increasingly important tool in the field of marine conservation and management [22]–[25]. In addition to prediction of single species distributions, maximum entropy modelling has also been used on species assemblages [26] or functional guilds [27] when broader conservation insights are desired.

Our study makes three main contributions. 1. We demonstrate how presence-only predictive habitat models can be used to gain rapid inference on the habitat requirements of species of conservation concern in situations of limited data. 2. Through international collaboration and use of multiple lines of empirical evidence, we present the first cumulative perspective on essential habitat for top marine predators across seasons in the Channel-North Sea basin. 3. In the context of much needed cooperative spatial planning policies, our study spotlights some critical data gaps that continue to hinder management on an ecosystem level. We propose the steps necessary to gain a deeper understanding of the place of megafauna in this socio-economically important region of the North-East Atlantic.

## Materials and Methods

### Ethics statement

The described study was conducted as a meta-analysis of archived marine megafauna observations in European waters. Each of the individual, prior research programs was carried out under European regulation regarding the use of wild and stranded marine megafauna for scientific and conservation purposes in the United Kingdom and France. No permits were required for the observations of wild marine megafauna. Stranded animals found at-sea and along European coasts by several organisations were considered, however no biological samples were used for this study. The collection of stranded animals is delegated to regional or national organisations under the permission of different institutions. In the United Kingdom, the Department of the Environment, Food and Rural Affairs is the authority to remove stranded animals for post-mortem examination. In France, this authority comes from the Ministry for Environment, Sustainable Development and Ecology.

### Study area

**Figure 1:**
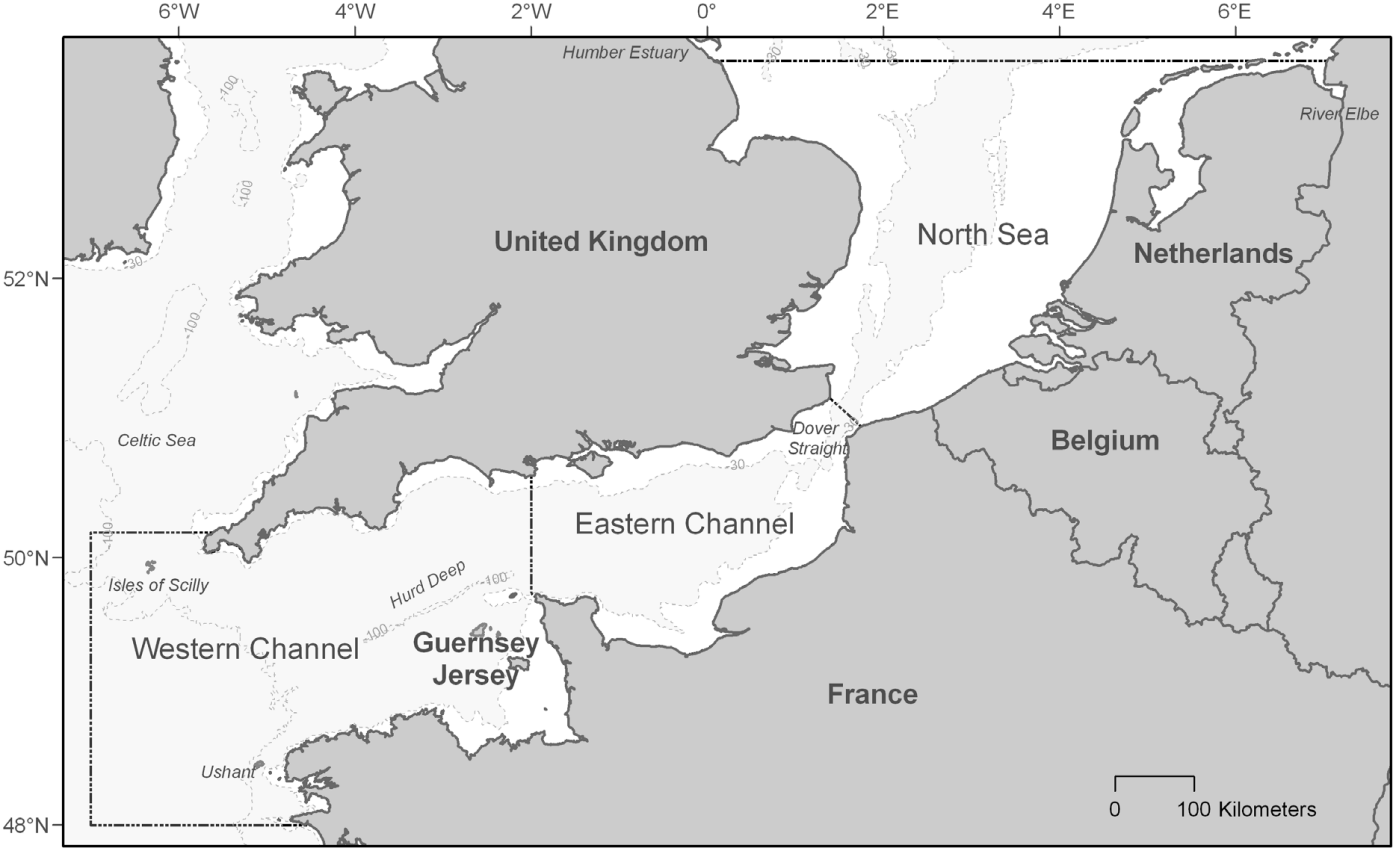
Study area. The study regions of the English Channel and North Sea indicated by dashed lines. Depth contours (30 and 100 m) in light grey. Areas <30 m are white.

The area of investigation was defined by the CHARM III (CHannel integrated Approach for marine Resource Management) project to include the whole of the English Channel and southern bight of the North Sea. This region covers approximately 180,000 km^2^ between the Humber estuary (53°30′N, 0°4′E) on the English coast, the mouth of the river Elbe (53°30′N, 7°15′E) that sits at the northern tip of the Dutch coastline, the waters surrounding Ushant island (48°0′N, 7°0′W) off the French coast of Brittany, and the Isles of Scilly (50°10′N, 7°0′W) southwest of England's Cornish coastline (Figure 1). The United Kingdom, Channel Islands (Guernsey and Jersey), France, Belgium, and the Netherlands claim exclusive economic rights to marine resources within this zone making it a hotspot of human activity.

These narrow and shallow marginal seas of the North-East Atlantic Ocean lie entirely on the European continental shelf where water-mass circulation is driven by tidal, wind, and density forcing [3]. The Western Channel has a notably deeper basin (≤100 m) that steeply slopes from the shoreline to a narrow undersea valley (the Hurd Deep) at its centre, whereas the shallow littoral zones of the Eastern Channel gradually widen approaching Dover Straight, and the Southern North Sea is distinctly shallower with most waters ≤30 m (Figure 1). The sea temperatures of entire region are influenced by the North Atlantic Drift and Gulf Stream Current warm water intrusions. A variety of frontal zones form in Western Channel sites that stimulate productivity [28]–[29], particularly around archipelagos [30]–[31], prominent coastal features such as headlands [30], and thermal and density boundaries [28]–[29], [32]. High freshwater flow from rivers and coastal runoff reduce salinities and increase primary productivity to a much greater degree in the North Sea. Seasonal cycles have a significant influence on the region's physical and biological oceanography [33]. These dynamics regulate the availability of the forage base and are reflected in the natural abundance, composition, and distribution of marine organisms from primary producers to top predators. A diverse assemblage of marine megafauna inhabits these waters, but many of these species are of conservation concern, both regionally and globally.

### General approach

We used methods described in Phillips et al. [20], [34] to perform multivariate analysis of sightings data organised by species and family groups. We also explored spatial and temporal trends of strandings (cetaceans and marine turtles only) to maximise insight into the probability of presence of these animals in the region.

### Data (requirements and processing)

For the purpose of this study, we used archives of geographically- and temporally-referenced marine megafauna datasets that were made available at no cost from data providers, as well as new data that were collected under the CHARM III project. Generally, these included sightings and strandings records from dedicated and opportunistic observer programmes operating from land, sea, or air by government, academic, and private research institutions in France and the United Kingdom. The vast majority of datasets did not have associated measures of observer effort, and even for those that did include effort information, metrics of effort correction were not directly comparable. Therefore, all data were treated as presence-only. Each dataset was individually pre-processed to remove errors and uncertainties (e.g. records collected in adverse weather, or in Beaufort sea states >3), to remove records that were not identified at least to the level of taxonomic family, to crop larger-extents to the bounds of our study area, and to standardise formatting of location, date, and taxonomic organisation. Additional fields were added to distinguish guilds, seasons, and to classify observations as either live or dead animals. We assembled the cleaned, presence-only datasets into either a single sighting or stranding database. Strandings data were not used in predictive models, but were used rather for complimentary descriptive analyses of species' occurrence trends and composition.

Due to large variation in the number of records and in the spatial and temporal resolutions of the individual datasets, we choose to analyse data in 4 km grid cells at the level of seasons pooled across years. We believe this was an appropriate decision to achieve a generalised picture of the distribution of long-lived, migratory animals in the extent of our study area, and because initial inspection revealed larger seasonal variation in the spread of observations than among years for a given species or group. Sightings data were brought into the geographic information system (GIS) software ArcGIS 9.3.1 [35], converted into the European Albers Equal Area Projection, and interpolated with various environmental surfaces using Marine Geospatial Ecology Tools (MGET) v.0.8a28 [36]. Point data were converted into binary rasters (0.25° resolution) that represented umbrella groups of megafauna (large pelagic fishes including sharks, cetaceans, pelagic seabirds, marine turtles, and pinnipeds). An index of biodiversity was then created by summing together these individual grids in raster calculator resulting in scores ranging from 0 (no group present) to 5 (all groups present).

The background environment was defined by generating 10,000 random points within the study area using Hawth's Tools [37] and then sampling all environmental surfaces (see below); dynamic variables were sampled from seasonal averages (spring = March–May; summer = June–August; autumn = September–November; winter = December–February). Previous studies have shown that differences in spatial bias between species and background data can result in inaccurate models [34]. The distribution of our point samples was clustered; to account for the spatial bias in species' location data, background sampling points were randomly generated into the 25^th^, 50^th^, 75^th^, 99^th^, and 100^th^ volume density contours of the sightings data. The use of target-group background data (i.e. matching spatial biases) has been known to considerably improve performance of species' niche modelling [34].

Cetaceans, seabirds, marine turtles, and large pelagic fishes may differentially select habitats in relation to *inter alia* environmental conditions, topographic features, and prey availability [38]–[42]. We obtained oceanographic data from a variety of freely-available sources for the years 2002–2011 and re-sampled raster data into 4 km grid cells in GIS with MGET for use in predictive habitat models. Briefly, monthly, 4 km global oceanographic AVHRR (Advanced Very High Resolution Radiometer) SST (sea surface temperature) and MODIS (Moderate Resolution Imaging Spectroradiometer) chlorophyll *a* concentration data from NOAA (National Ocean and Atmospheric Administration) satellite imagery were downloaded and converted to 3-month-mean climatologies and clipped to our study area. We interpolated NOAA's World Ocean Atlas monthly surface salinity data to generate seasonal mean rasters. The choice to derive seasonal climatologies was made to make the best use of marine animal data (i.e. changes in occurrence and sample size). S2004 bathymetric data [43] were used to construct a continuous raster surface of ocean bottom slope (measured in °) using the “slope” function, and to identify the continental shelf break (200 m isobath). A distance to shelf break raster was then generated using a “Euclidean distance” function. In the same manner, we created a distance to shore layer using ESRI's high resolution shoreline shapefile. To provide an indication of the regional availability of primary consumers, monthly zooplankton data were obtained from Continuous Plankton Recorder (CPR) surveys conducted in the study region [44] for years that best coincided with available satellite imagery and marine predator data (2002–2010). Copepod and gelatinous zooplankton presence-only records were used to create seasonal (3-month) interpolated surfaces of probability of occurrence [45].

### Predictive modelling

We used presence-only species' distribution models to develop seasonal habitat probability (probability of presence on a scale of 0–1 where values of 0 indicates low likelihood of animal being present and 1 high likelihood of animal being present) and suitability maps (habitat or non-habitat) using the software Maxent v.3.3.3k. Maxent estimates a species' distribution within a given area by finding the probability distribution of maximum entropy based on the constraint that the expected value of each background environmental variable should match its empirical average [20]. This method is preferable for small sample sizes, allows the combination of diverse data sources or with biases in their spatial coverage, and selects the most important environmental variables driving the species' distribution. Correlated variables, however, can confound interpretation of variable importance in model results; therefore prior to maximum entropy modelling, we calculated pair-wise Pearson's correlations of all environmental variables to determine which variable(s) should be eliminated from the models.

Separate Maxent models were run by season for each species or assemblage using cross-validation to split training (i.e. model building) and test (i.e. model validating) data into equal-sized folds due to small sample sizes (Table S1). We ran 25 replicates of each model using default parameters to produce spatial predictions of the mean probability of presence for each megafauna species/group accompanied by a series of diagnostic outputs. The mean minimum training presence logistic threshold, defined as the threshold that includes all areas that are at least as suitable as those where the species is known to occur in the training dataset, was used to re-classify these results into habitat suitability maps. We evaluated each Maxent prediction using the AUC (area under the receiver operating characteristic curve) threshold-independent metric, which assesses model discriminatory power by comparing model sensitivity (i.e. true positives) against model specificity (i.e. false positives) from a set of test data [46]. The AUC scale ranges from 0–1; an AUC score of 0.5 indicates that the model was no better than random at discriminating habitat, while higher scores indicate improving accuracy. It should be noted however that AUC values are typically lower for species with wide distribution ranges [34], such as all species in this study. We used jackknife analysis of AUCs to estimate individual variable contributions to each of the resulting models.

## Results

### Overview

A total of 78 species of megafauna were observed in the study area (Table S2). More than half of these were pelagic seabirds (N = 43), followed by cetaceans (N = 20, although 4 of these occurred primarily as strandings), pinnipeds (N = 6, 4 only as strandings), sharks (N = 4), marine turtles (N = 4, 3 primarily as strandings), and large pelagic teleost fish (N = 1). A total of 63,478 out of 269,756 observations (119,924 occurring in our study area) of marine megafauna were available for analysis after our rigorous filtering procedure. In the final database, 9% of records were strandings and 91% were sightings. Together these represent the best available data on marine megafauna in the Channel-North Sea basin, originating from 30 different datasets provided by 16 different organizations (4 France, 11 United Kingdom) over a period of 250 years, albeit most observations were from the last 25 years. A few marine turtle records dated back to the mid-1700s, but the majority of the data represented cetacean and seabird sightings from the mid-1990s to 2011. With the exception of 4 species, all are listed on the International Union for Conservation of Nature (IUCN) Red List for global conservation concern, and of these, 63% require consideration by one or more European conservation legislations (95% of cetaceans, 50% of sharks, 100% of marine turtles, 40% of pinnipeds, and 49% of pelagic seabirds) (Table S2).

We produced 36 niche models of individual or grouped species within a season. In all cases but one, models produced acceptable to outstanding habitat discrimination [47] with AUC values ranging 0.69 to 0.92. For the purpose of this paper, we present the results of select examples to demonstrate the performance of the maximum entropy modelling across a diversity of megafauna.

### Species Examples

#### Harbour Porpoise

**Figure 2:**
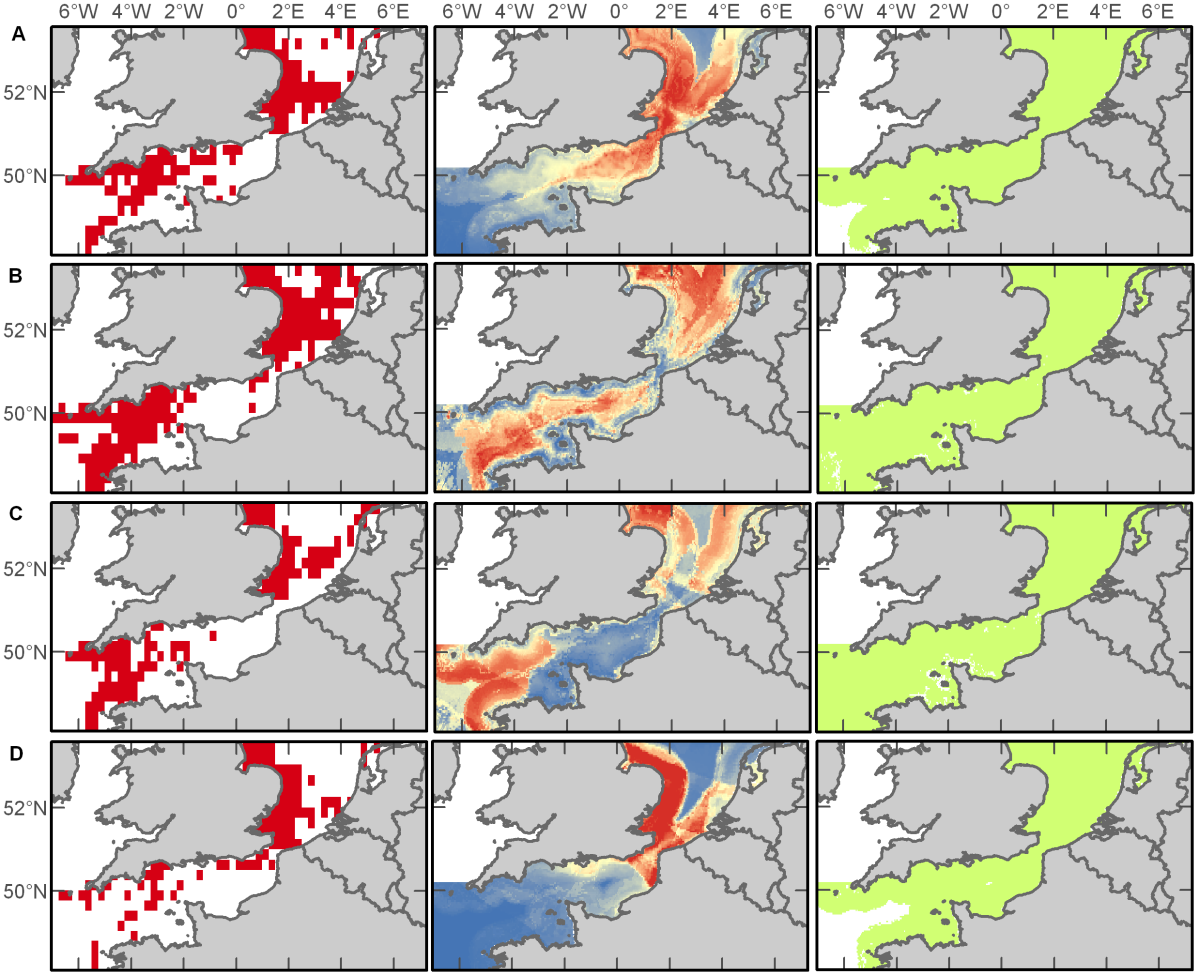
Harbour porpoise distributions. Empirical observations (left column) and Maxent predictions of probable (middle column) and suitable habitat (right column) in spring (A), summer (B), autumn (C), and winter (D). Warmer colours in middle plots indicate a higher probability of presence.

Despite its small size and inconspicuous colouration, the harbour porpoise (*Phocoena phocoena*) was the most commonly sighted cetacean (59% of records) in the database. These animals occurred in all three zones of the study area (Western Channel, Eastern Channel, and North Sea) throughout the year, but demonstrated seasonal expansions and contractions in their distribution (Figure 2). In jackknife analysis of environmental variable importance within Maxent predictions, distance to the continental shelf swamped all other contributions in both spring (86%) and winter (70%) harbour porpoise distribution models, while distance to shore (49%), SST (18%), and bathymetry (17%) were the greatest contributions in summer, and distance to shore (40%) and SST (30%) were greatest in autumn (Figure S1). Among the seasonal models, the winter prediction had the highest AUC score showing excellent discrimination of harbour porpoise habitat followed by the spring, summer and autumn, each performing acceptably (Figure S2). The resulting spatial representations of the maximum entropy modelling suggest that the entire study area is essentially suitable habitat for harbour porpoises, but the probability of occurrence varies with the seasons; based on our model predictions, this shy species is more likely to be encountered in the Southern North Sea during the winter and spring, move throughout the Channel in the summer, and then retract to the North Sea and Western Channel by the autumn (Figure 2).

#### Leatherback Turtle

**Figure 3:**
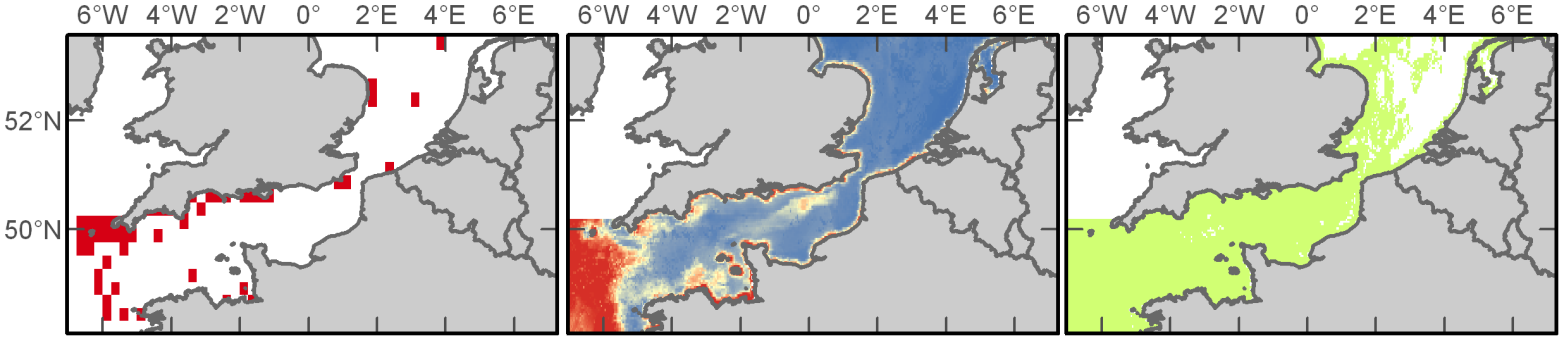
Leatherback turtle distributions. Empirical observation and Maxent prediction of probable and suitable habitat (left to right) in summer. Warmer colours in middle plots indicate a higher probability of presence.

Leatherback turtles (*Dermochelys coriacea*) are the largest species of marine turtles and the only one to regularly visit higher latitude waters, albeit rarely spotted within the study area. The vast majority of the sightings (86% of records) occurred in the Western Channel, followed by the Eastern Channel (8%) and North Sea (6%) (Figure 3). Although small numbers of leatherback turtles were documented year-round, we were only able to produce a prediction for their summertime distribution due to insufficient sample sizes in other seasons (winter N = 2; spring N = 1; autumn N = 23). Distance to shore (49%) and chlorophyll *a* (34.5%) were the most important variables contributing to this model (Figure S3), which resulted in excellent habitat discrimination (Figure S4). The predictive model suggests that these primarily oceanic animals are restricted to the South-Western Approaches of the Channel (Figure 3).

#### Basking Shark

**Figure 4:**
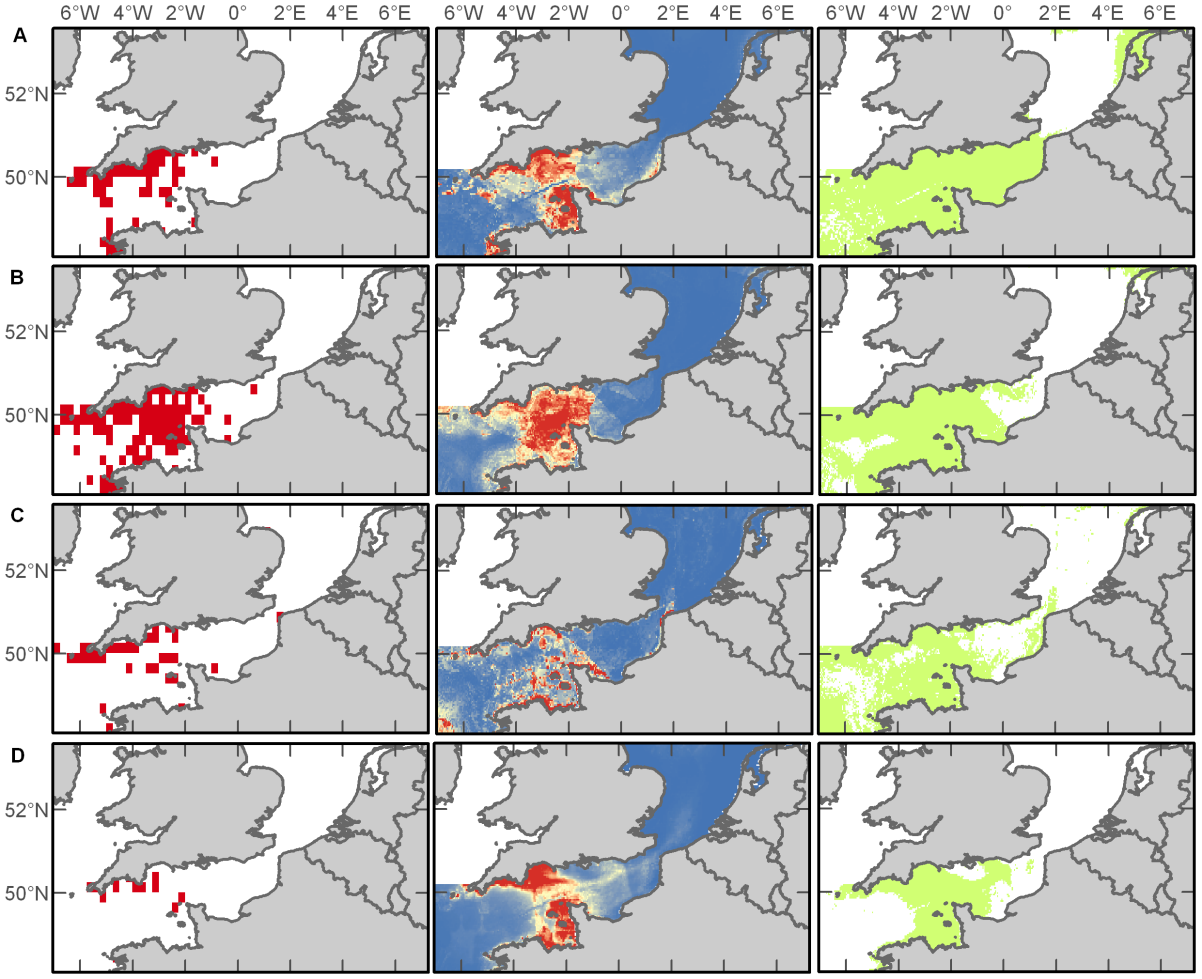
Basking shark distributions. Empirical observations and Maxent predictions of probable and suitable habitat (left to right) in spring (A), summer (B), autumn (C), and winter (D). Warmer colours in middle plots indicate a higher probability of presence.

Basking sharks (*Cetorhinus maximus*) are among the largest marine species and one of the few zooplanktivorous sharks. Peak sightings of these animals were recorded during the summer months and with fewest sightings during winter. The majority of observations (>99% of records) of these animals were from the Western Channel (Figure 4). In analysis of variable importance, bathymetry (35%) and distance to shore (30%) were most important during spring, distance to shelf (57%) and salinity (17%) during summer, distance to shelf (38%) and chlorophyll *a* (35%) during autumn, and chlorophyll *a* (35%), salinity (23%), and bathymetry (22%) during winter (Figure S5). Models of basking sharks resulted in the best performance of any species in this study with outstanding habitat discrimination for the spring and autumn predictions and excellent power for summer and winter (Figure S6). It appears that much of the English Channel provides suitable habitat for basking sharks throughout the year, but that their presence is concentrated in the Western Channel (Figure 4).

#### Northern Gannet

**Figure 5:**
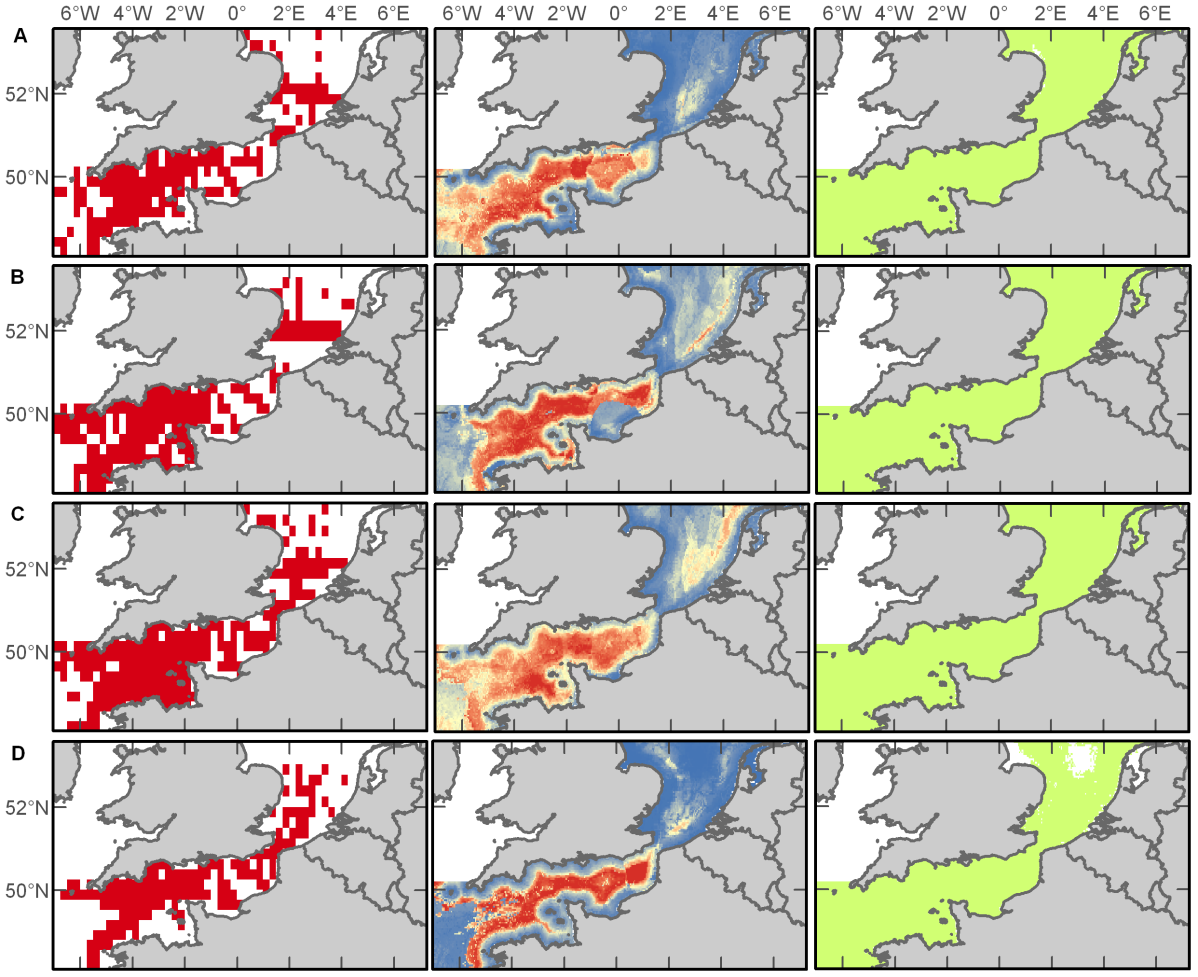
Northern gannet distributions. Empirical observations and Maxent predictions of probable and suitable habitat (left to right) in spring (A), summer (B), autumn (C), and winter (D). Warmer colours in middle plots indicate a higher probability of presence.

More than half of the world's Northern gannets (*Morus bassanus*) breed along the coasts of France and Britain, and can be found year-round within the study region. The at-sea sightings of these pelagic seabirds occurred mostly in the Western Channel (92% of records), although gannets were also recorded in the Eastern Channel (5%) and Southern North Sea (3%) (Figure 5). Distance to shore emerged as the most important variable contributing to all seasonal models (spring through winter 63%, 49%, 34%, 63%, respectively), although salinity was also important in the autumn model (33%) (Figure S7). The winter model produced excellent habitat discrimination and other seasons performed acceptably (Figure S8). The entire study area emerged as suitable habitat; while at-sea gannets are predicted to be most likely to occur in the English Channel, particularly off the Cotentin Peninsula (Figure 5).

### Family Group Examples

#### Dolphins

**Figure 6:**
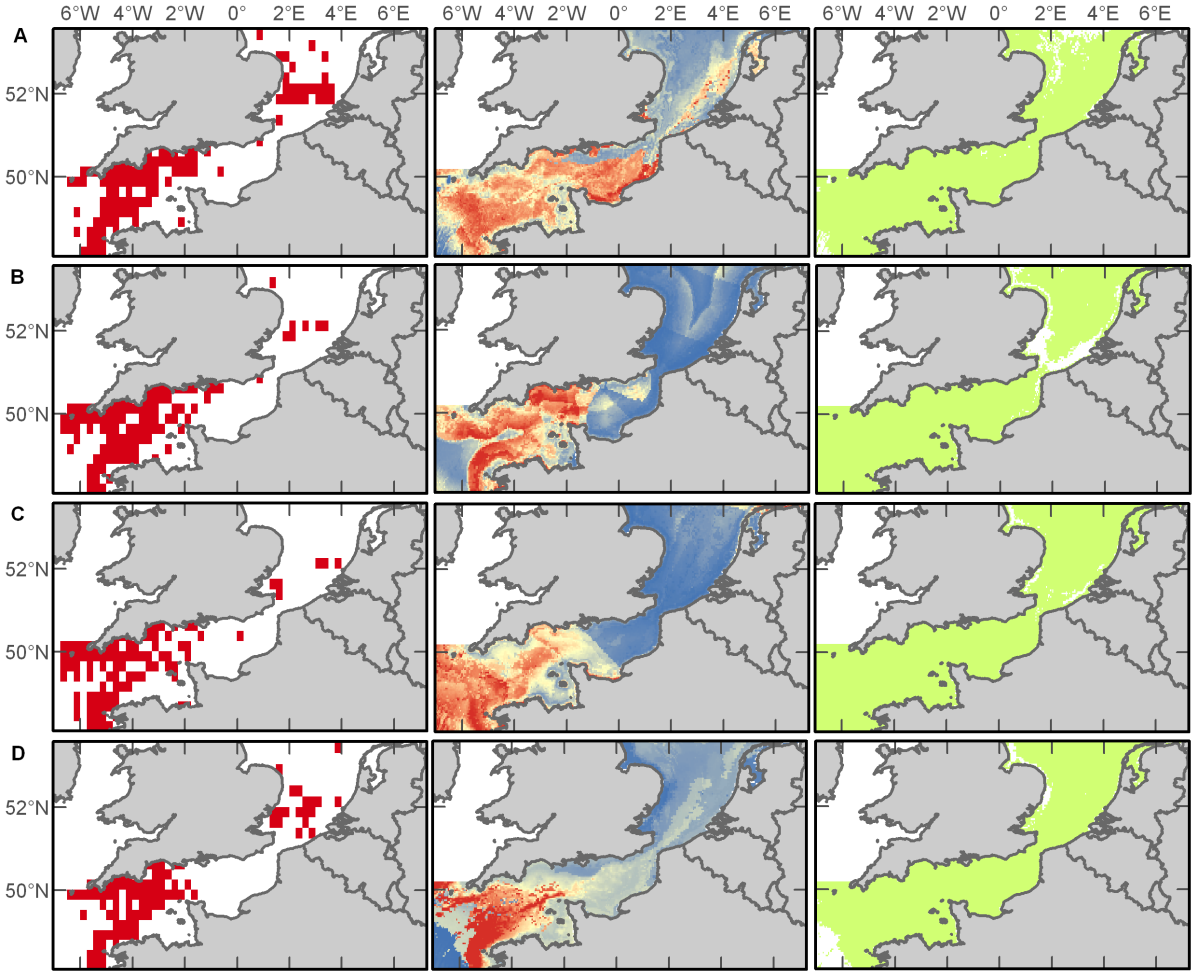
Dolphin distributions. Empirical observations and Maxent predictions of probable and suitable habitat (left to right) in spring (A), summer (B), autumn (C), and winter (D). Warmer colours in middle plots indicate a higher probability of presence.

Dolphins, together with blackfish (i.e. pilot, killer, and false killer whales), are odontocete members of the cetacean family Delphinidae. As a group they are of interest because they are wide-ranging and easily recognisable marine predators that frequently co-occur in mixed species assemblages. In our study, this group was represented by 6 species including short-beaked common (*Delphinus delphis*), bottlenose (*Tursiops truncatus*), Risso's (*Grampus griseus*), white-beaked (*Lagenorhynchus albirostris*), striped (*Stenella coeruleoalba*), and Atlantic white-sided (*Lagenorhynchus acutus*) dolphins. For reasons of sample size and application within the context of our over-arching goals, we present a combined analysis of species. Curiously few (2% of records) dolphins have been observed in the Eastern Channel, little more (3%) in the North Sea, whereas nearly all records (95%) occurred in the Western Channel irrespective of season (Figure 6). In models analysing spring conditions, SST (40%) and chlorophyll *a* (30%) contributed the most to the predicted distribution of dolphins, while for summer it was salinity (44%) and distance to shore (21%), for autumn it was distance to shelf (51%) and bathymetry (39%), and in the winter model bathymetry (55%) and chlorophyll *a* (46%) contributed nearly equally (Figure S9). Parameters important in the dolphin models were highly varied according to season yet performed equally well in discriminating habitat among seasons (Figure S10) and produced similar spatial distributions. Maxent results consistently predicted that dolphins would be more likely encountered in the English Channel, despite classification of suitable habitat throughout the entire study area (Figure 6). Model performance was slightly higher (AUCs 0.82–0.83) in models of bottlenose dolphins run as a single species (not shown); their predicted distributions were notably more constrained with less of the North Sea being characterised as suitable habitat and even the Channel being unsuitable during the autumn.

#### Auks

**Figure 7:**
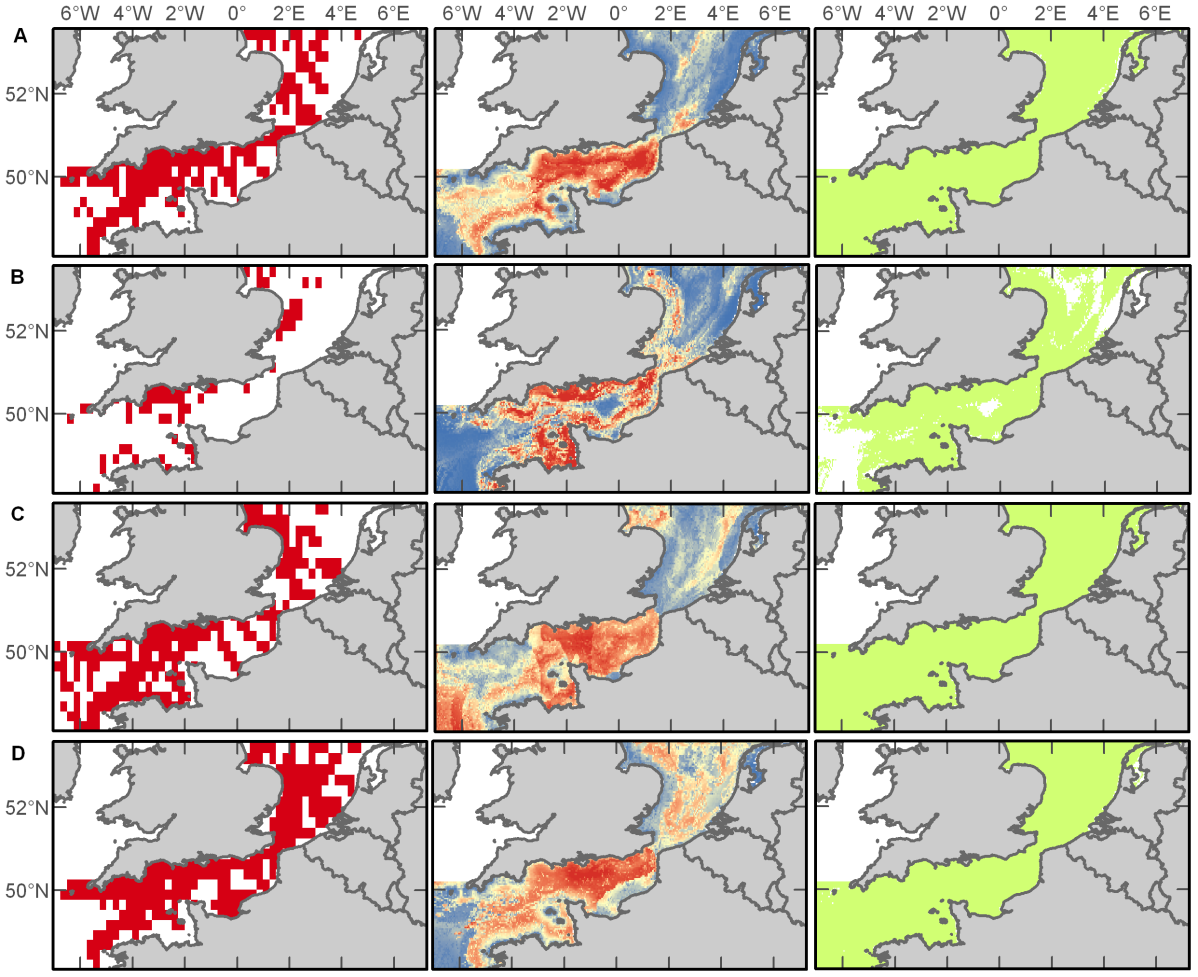
Auk distributions. Empirical observations and Maxent predictions of probable and suitable habitat (left to right) in spring (A), summer (B), autumn (C), and winter (D). Warmer colours in middle plots indicate a higher probability of presence.

Auks are members of the Alcidae family of seabirds and in our study region include common guillemots (*Uria aalge*), razorbills (*Alca torda*), and Atlantic puffins (*Fratercula arctica*). Auks occurred throughout the study area throughout the year with the most records in the Western Channel (65% of records) followed by the Eastern Channel (20%) and Southern North Sea (15%), although sightings precipitously declined overall in the summer months and were constrained primarily toward the coast of England (Figure 7). Variables that emerged as important in predictive habitat models differed greatly between seasons (Figure S11), yet resulted in acceptable (for summer and winter models) and excellent (for spring and autumn models) habitat discrimination (Figure S12). For the spring model, the variables distance to shore (52%) and SST (20%) contributed the most to the resulting prediction. For summer, it was slope (49%) and distance to shore (22%). For autumn, it was salinity (44%) followed by distance to shore (20%). For the winter model, distance to shore (41%) and chlorophyll *a* (33%) contributed the most to the model prediction. Maxent predicted that the entire study region is suitable for auks, with a few exceptions during the summertime, but the highest probability of occurrence is in the Channel, from east to west (Figure 7).

### Strandings

**Figure 8:**
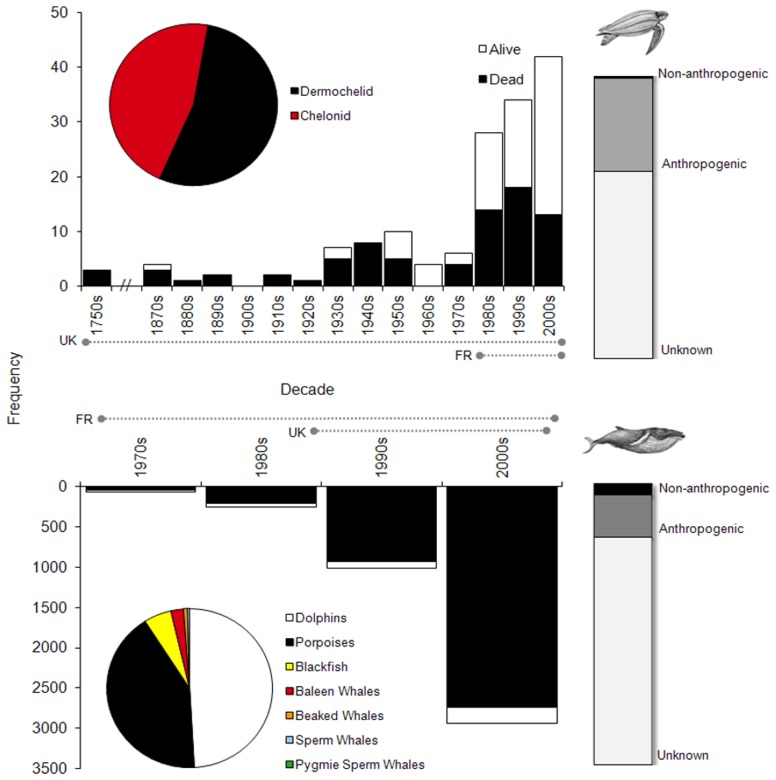
Stranding summaries. Graphical summaries of strandings' composition (pie chart), effort-uncorrected trends (country-specific, time-series duration indicated by dotted lines along x-axis) and status (main bar chart), and cause (bar chart on far right) for marine turtles (top) and cetaceans (bottom).

Stranding records of cetaceans (1971–2009) and marine turtles (1750–2009) in the study increased over time, with nearly 30 times more cetacean records in the database than turtles despite the shorter time-series (Figure 8). There were no data available to correct for observer effort. The proportion of live stranded animals was consistently low across years for cetaceans but increased for marine turtles (Figure 8). Because determining the cause of stranding is complicated and dependent on many factors (e.g. physical state of the animal upon discovery, access for retrieval/post-mortem examination, funding limits, etc.), the majority of cases (66% for marine turtles, 81% for cetaceans) were categorised as of “unknown” cause. This category combined animals that received post mortem examination, whereby the cause of death was not determined or determined but could not be definitively placed into one of the following categories, and those that did not. For the remaining stranding events, 33% of turtles and 15% of cetaceans were determined to be as a result of “anthropogenic” causes (i.e. bycatch, entanglement, ship strikes, direct killing), whereas 1% of turtles' and 4% of cetaceans' strandings were determined to be from “non-anthropogenic” causes (i.e. disease, poor condition/starvation, non-specific physical trauma) (Figure 8).

Although documented in near equal numbers (Figure 8), Dermochelid turtle strandings (i.e. leatherbacks) occurred most frequently in the warmer months of autumn and summer, while Chelonid turtle strandings (i.e. loggerhead *Chelonia mydas*, Kemp's ridley *Lepidochelys kempii*, and green *Chelonia mydas*) peaked in the colder spring and winter months (Figure S13A). The majority of marine turtle stranding events (82% of records) were in the Western Channel, followed by the Eastern Channel (13%) and North Sea (5%) (Figure S13A). Among the 20 species of cetaceans in the stranding database, dolphins made up 49% of the records, porpoises 42%, blackfish 5%, baleen whales 2%, beaked whales 1% and sperm and pygmy sperm whales made up <1% each (Table S2, Figure 8). Considered together, cetacean strandings were greatest in the winter and spring and lowest in the summer and autumn, however there were differences among taxonomic groups (Figure S13B). For instance, baleen whale strandings, although low in number, were relatively equal in all seasons, while peak blackfish strandings occurred in the autumn and winter, and the rare beaked whale strandings were highest in summer. The Western Channel accounted for 63% of cetacean stranding records, followed by 19% in the North Sea and 18% in the Eastern Channel; this pattern was similar across seasons (Figure S13B).

### Megafauna hotspots

**Figure 9:**
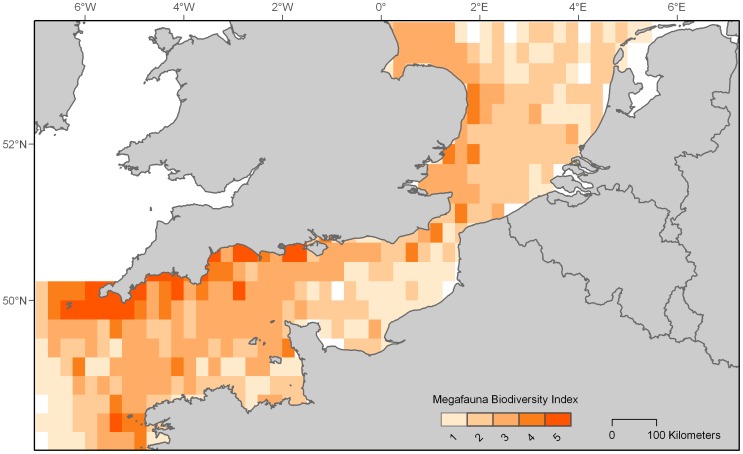
Index of marine megafauna biodiversity. Colour scale indicates number of umbrella groups (large pelagic fish, cetaceans, marine turtles, seabirds and pinnipeds) present per cell where warmer colours point to hotspots.

Biodiversity index scores were high in the Western English Channel, with particular hotspots around the southern tip of the United Kingdom, low in the Eastern Channel, and intermediate in the North Sea (Figure 9). Diversity east of the prime meridian was driven by seals, pelagic seabirds, and porpoises.

## Discussion

The description of an organism's niche is a fundamental concept in ecology [48] and one of the first tiers sought in species' conservation regimes. It is often a difficult goal to achieve for marine fauna due to sparse information, particularly in the case of pelagic animals that differentially exploit vast expanses of ocean throughout their life cycle. We undertook a heuristic approach to this problem. Using hierarchical habitat models, we were able to produce spatial distribution maps of key marine predators, which could be a valuable instrument for those authorities confronted with the task of designating marine protected areas in a sea bustling with human activity.

### What have we learned?

#### Biodiversity

Despite the coarse scale of our analyses, we now have the first description of the ecological footprint for marine megafauna specific to the Channel region. Our combined database documented considerable biodiversity encompassing 20% of the world's cetacean species and 10% of global seabird species. The variety and importance of roles these top predators play within trophic structures has been rarely appreciated or applied in ecosystem-based analyses here or elsewhere [49], despite historic examples that echo ecosystem changes induced by dramatic population declines of key species [50]–[53]. Considered together, marine megafauna feed across the entire trophic spectrum, but individually, many of these species are more specialised foragers occupying unique niches of the marine foodweb. Nevertheless, they frequently co-occur and identification of hotspots for biodiversity is a top conservation priority [54]. In the context of our study area, the Western Channel surfaces repeatedly among top predator species as a key area, with the greatest diversity occurring near promontories and islands off Cornwall and Brittany. Our next logical step then is to capture and conserve the underlying biological and physical processes that form this biodiversity at the appropriate scales [55].

#### Conservation Tools

The use of maximum entropy modelling proved valuable not only for identifying important areas, but also for beginning to elucidate the species-environment relationships that shaped the spatial patterns in the data. Although it is unsurprising that species-specific data produced more accurate models, the use of species assemblages or guilds may be a practical option when the goal is not to learn more about particular species' ecologies, but to encompass habitat or features that will benefit a larger community. In this study, the use of seasonal models was compelling due to the itinerant nature of marine predators and to the periodicity in the biotic and abiotic environment. These very attributes have raised concern over the efficacy of ocean zoning [56]. Nevertheless, marine reserves are considered the best tool for ecosystem-based marine conservation and management [57] even for pelagic species [58]–[59]. Species with critical habitat protection are twice as likely to exhibit population recovery [60], but for long-lived, marine species with life histories that rely upon high adult survival, large reserves are required to produce an effective demographic response [61]. In European waters, the Natura 2000 initiative seeks to establish a network of protected sites to preserve marine biodiversity. While the concept is still evolving, dynamic marine reserves could provide desirable flexibility in the timing and placement of protected areas, which may improve reserve performance [62] and accommodate ephemeral oceanographic features that aggregate species [63].

#### Species' niche

Marine megafauna occurring in temperate European waters are comprised of resident populations as well as migrating individuals and include both near-shore and pelagic species that rely on this shelf habitat for differing life history functions. Our study area, for instance, represents core foraging grounds for internationally important numbers of breeding northern gannets [64]–[66], as well as important sites for overwintering gulls, auks, great skuas (*Stercorarius skua*), and Balearic shearwaters (*Puffinus mauretanicus*) [67]–[69] and migrating Atlantic puffins and Manx shearwaters (*Puffinus puffinus*) [70]–[71]. Grey seals (*Halichoerus grypus*) also are a resident species that occupy most of the Western Channel, the English side of the Eastern Channel, and a small area of the Southern North Sea bight to forage and shuttle [72]–[73] between their rookeries in northwest Brittany [74], Cornwall and the Isles of Scilly [75], and Solent Harbour [73] and their nearby haul-out sites on the French coast, the Isles of Scilly, Cornwall, Wales, the Channel Islands and Norfolk [72], [74]–[75]. Small populations of bottlenose dolphins reside off of Cornwall [76] and along the French coast primarily around the Cotentin peninsula [77], some of which undertake long distance movement among the British Isles [76], [78]–[79]. Seasonal occurrence, on the other hand, is more evident in pelagic species that make forays into the Channel, but are less frequently observed. Leatherback sea turtles, for example, are an oceanic species that appears in the region primarily during the summer coincident with the timing for arrival to well known northern foraging grounds in the Atlantic [80]. Similarly, other large, enigmatic pelagic predators such as basking sharks, ocean sunfish (*Mola mola*), minke whales (*Balaenoptera acutorostrata*), Risso's dolphins, killer whales (*Orcinus orca*), and long-finned pilot whales (*Globicephala melas*) approach the coastal waters of the Western Channel during a few months [77], [81]–[84] presumably in pursuit of food.

Although the role of episodic small-scale features such as fronts and eddies are difficult to capture with the resolution of our sightings data, it is noteworthy that the results of our spatial predictions for many species show increased encounter probabilities in the Western Channel where fronts often occur (see description of study area). Associations of basking sharks [38], [84], ocean sunfish [85], northern gannets [64], [86], leatherback turtles [45], [87]–[88], bottlenose dolphins [89], and minke whales [90] with frontal zones, topographic and bathymetric features that stimulate frontal activity (i.e. headlands, straights, and deepwater drop-offs), or dense aggregations of planktonic organisms facilitated by fronts have been documented in the regions surrounding our study area suggesting that these oceanographic features do indeed play an important role in the distribution of a wide range of marine megafauna.

Other species, which forage more diversely on fishes, exhibited more even spatial distributions, but marked seasonal variation. Auks, for example, form large breeding aggregations along coastlines in the west [91], but our findings also highlight the importance of the shallower waters in the east of the region for these diving avian predators. The significance of these waters is also evident for dolphins and porpoises [81]–[82]. Environmental characteristics that influence the distribution of small cetaceans appear to differ geographically, and by season as we found in this study; nevertheless factors such as depth, slope, proximity to the coast, tidal state, and SST repeatedly surface as being important habitat features [22], [89], [92]–[101].

While it is clear from this study that the English Channel and southern bight of the North Sea support numerous species of marine megafauna today, a review of previous species accounts suggests that there have been some noteworthy changes over time – albeit as in most situations baselines are lacking. The occurrence of white-beaked dolphins [77], [82], harbour porpoises [102]–[104], basking sharks [84], Balearic shearwaters [67], and minke whales [82], [105] appear to have increased in frequency in our data reflecting either population increases/recovery or expansions/re-expansions in the area; however this could also be indicative of increased observer effort. On the contrary, there is concern that certain bottlenose dolphin populations may be in decline [76], [101].

#### A healthy dose of caution

This study has shown that there is a clear, over-arching spatial structure in the distribution of marine megafauna within the Channel-North Sea basin and that has been corroborated through multiple lines of evidence. Nevertheless, this tells us nothing about the true density of animals occupying these habitats. Our modelling exercise produced ecologically plausible results, but the resulting niche descriptions were limited by the set of available environmental parameters, did not account for prey availability in the majority of models, and were artificially constrained by the bounds of the study area. Despite our efforts to be comprehensive, we recognise that our data has inherent biases and we took measures to account for these in our analytical approach where possible. Trends in strandings data most certainly reflect an increase in observer effort, but may also include changes in the population as well as frequency of human interactions. Not all stranded animals received full post-mortem examination and our representation of the proportional cause of stranding may contain inaccuracies due to indirect sources of mortality such as exposure to contaminants or environmental stressors. Our analyses were necessarily coarse in resolution due to missing data, but the spatial and temporal scales used are relevant to the long-lived and far-ranging species examined. Even so, the seasonal approach we employed certainly masks temporal variation in species occurrence and dynamic environmental processes and the hotspot regions we highlighted are far larger than most MPAs around the globe [106]. The patterns we have presented therefore represent a generalised view across time within a particular focal region and are meant to direct further efforts.

### What still needs to be done?

As part of the process to achieve a broad-scale perspective on marine megafauna in the English Channel, we were able to identify data deficiencies that continue to impede an ecosystem management approach. For example, precise measures of abundance of most top marine predators are lacking. To achieve this goal requires large-scale, multi-platform, effort-related surveys across the study area to be carried out across seasons and preferably repeated over sufficient time periods to be able to detect temporal trends [107]. With exception of a few programs [67], [105], [108]–[109] this has not been attempted systematically in Europe. The use of platforms of opportunity have been explored to fill that need [97], [104], however these surveys are spatially patchy and serially autocorrelated – factors that do not conform to the necessary experimental design needed for density estimation techniques [110]–[111]. Attempts have been made within the CHARM III project to supplement effort into un-observed seas and use estimates of relative abundance to make Channel-wide inferences. However there is a critical need for comparability estimates between platforms with respect to sightability. Moreover, statistical theory assumes that samples are taken synoptically, which was logistically untenable in the latter case. Previous surveys have most frequently focused on specific seasons – particularly summer – however, repeated measures in each area across the whole year are necessary to determine the nature of any temporal patterns in abundance.

Changes in top predator communities can have far-reaching and unexpected consequences in the trophic dynamics of an ecosystem [112]–[113]; however the functional role of megafauna in most systems is still poorly understood. Data on biomass and energetic requirements do not exist for many ocean giants for practical reasons, but reasonable approximation could be achieved through ancillary methods (i.e. use of captive animal records, strandings, 3D digital imagery). Spatially explicit data are required for the distribution of potential prey. Diet, isotopic, and bomb calorimetric studies are needed to identify prey species and quantify fractional importance with associated measures of energetic value. Within the English Channel, there is a spatial mismatch between data available on marine megafauna (Western Channel) and lower trophic levels (Eastern Channel). Therefore additional studies for top predators are needed in the Eastern Channel, while studies on pelagic and dermersal prey species need to expand into the Western Channel before realising a complete ecosystem network analysis.

Information on animal movements and local habitat use in this region is limited to a relatively few marine megafauna taxa over relatively short durations [38], [64]–[65], [74], [76], ; however such studies are critically needed in order to determine residency patterns, home ranges, and site fidelity within key sites. An increase in mark-recapture, photo-ID, and telemetry studies could address these questions. Moreover, presence-only analytical techniques offer the ability to integrate these types of data [22], [115] into meta-analyses that seek to elucidate important environmental forces and potential spatial conservation strategies.

Issues of scale need to be investigated to illuminate species-environment relationships [116]. For example, temporal dynamics may govern species distribution patterns in relation to episodic phenomena (i.e. meteorological drivers, ephemeral fronts), daily cycles (i.e. diurnal tides, diel vertical migrations), seasonal events (i.e. plankton blooms, water mass stratification, mixing and convergence), and decadal cycles (i.e. North Atlantic Oscillation). Long-term local ecological research is well represented for short-lived plankton [33], but ironically studies of long-lived marine vertebrates are typically short in duration. At present, stranding and public sightings data series are the only continuous, long-term source of information on marine megafauna in the area. In order to understand changes in top-predator species' distributions, long time-series are essential [117]–[118].

Understanding geographic and temporal trends in marine populations is therefore critical for contextualising anthropogenic impacts and developing effective and sustainable conservation management strategies [119]. Oceanic regime shifts, for example, are currently a serious concern [120]–[121]. In the face of a changing climate, it would be beneficial to forecast climate-driven scenarios using the current distribution patterns. The functionality to explore ecosystem state change already exists in Maxent [122] and would be a useful exercise for a range of sensitive species.

Integrating the species distributions with the distribution of potentially harmful activities, while obvious, is not straightforward. Spatiotemporal information on human behaviours may be sensitive, such as natural resource use (i.e. fisheries) or issues of national security (i.e. military training exercises), and are rarely forthcoming due to economic value and perception. Nevertheless, spatial overlap analyses are valuable tools for looking not only at risk, but also for evaluating planned or realised management measures [123]–[125]. Quantitative information on the nature and frequency of species' interactions with particular human activities is needed (i.e. bycatch, ship strikes, entanglement in marine debris, perturbation), however it must work both ways and as biologists we need to share our data in order to further conservation. Oceanographers set a laudable example by freely distributing their data to the masses (e.g. SST, primary productivity, altimetry).

## Conclusions

A meta-analysis of existing ecological datasets can be useful for highlighting both knowledge and gaps. Multi-level habitat models provide new avenues for identifying important places and environmental spaces of species and assemblages. Our study highlights an interesting conservation problem, which is to identify habitat preferences of highly cryptic and/or volant species, some of which are on the margin of their range. Although the study area is of lower habitat importance than the neighbouring regions for many of the top predators we examined (see [45], [67], [81]–[82], [109]), it encompasses the zone of highest human impact in the marine environment [2]. Whether anthropogenic activity drives this condition remains unclear, but maintaining ecosystem health and conservation of the current biodiversity in the region is mandated by marine policies [1]. The Western English Channel emerged as a hotspot for marine megafauna diversity – particularly in regions that produce frontal activity – and a network of marine reserves placed there would protect multiple species assemblages, functional guilds, and unique habitat and oceanographic features. It is also an area with high fisheries effort [126]. The Eastern Channel appears to maintain the least diversity. It is unclear whether this zone is the most degraded or whether it is a naturally empty place. Could this region gain then most from spatial planning? Several accounts suggest the southern bight of the North Sea is experiencing a recovery/re-expansion of top predator populations [102]–[103], [109]. If so, might this be low hanging conservation fruit? Where-ever ocean spatial planning initiatives proceed, marine megafauna require special consideration. Some species require both terrestrial and marine conservation, many move long distances as part of their general ecology, and most rely upon dynamic oceanographic features such that essential habitat could change with season or changes in their prey availability. Effective marine protected areas have already been established in the Channel that protect a large proportion of time at-sea for some species [74]. Such successes may produce further challenges – as populations increase, conflicts are likely to increase – therefore programs should incorporate adaptive management scenarios. Our study clearly demonstrates that integrated, cross-border information is an improvement into the previously nationally-focused accounts. Although this has been a good first step, several hurdles are still to be overcome in order to achieve an ecosystem-level understanding of how species, communities, processes, and humans can co-exist in a sustainable fashion.

## Supporting Information

Figure S1 Maxent results of jackknife analyses of the environmental variable importance for harbour porpoise predictions. Grey bars show the performance (in terms of training gain) of the global model without each variable and black bars show the influence with only that variable.
(TIF)

Figure S2 Maxent Receiver Operator Characteristic (ROC) curves and Area Under the Curve (AUC) values for training and test data for the harbour porpoise seasonal models.
(TIF)

Figure S3 Maxent results of jackknife analyses of the environmental variable importance for the leatherback prediction. Grey bars show the performance (in terms of training gain) of the global model without each variable and black bars show the influence with only that variable.
(TIF)

Figure S4 Maxent Receiver Operator Characteristic (ROC) curve and Area Under the Curve (AUC) value for training and test data for the leatherback turtle model.
(TIF)

Figure S5 Maxent results of jackknife analyses of the environmental variable importance for basking shark predictions. Grey bars show the performance (in terms of training gain) of the global model without each variable and black bars show the influence with only that variable.
(TIF)

Figure S6 Maxent Receiver Operator Characteristic (ROC) curves and Area Under the Curve (AUC) values for training and test data for the basking shark seasonal models.
(TIF)

Figure S7 Maxent results of jackknife analyses of the environmental variable importance for gannet predictions. Grey bars show the performance (in terms of training gain) of the global model without each variable and black bars show the influence with only that variable.
(TIF)

Figure S8 Maxent Receiver Operator Characteristic (ROC) curves and Area Under the Curve (AUC) values for training and test data for the gannet seasonal models.
(TIF)

Figure S9 Maxent results of jackknife analyses of the environmental variable importance for dolphin predictions. Grey bars show the performance (in terms of training gain) of the global model without each variable and black bars show the influence with only that variable.
(TIF)

Figure S10 Maxent Receiver Operator Characteristic (ROC) curves and Area Under the Curve (AUC) values for training and test data for the dolphin seasonal models.
(TIF)

Figure S11 Maxent results of jackknife analyses of the environmental variable importance for auk predictions. Grey bars show the performance (in terms of training gain) of the global model without each variable and black bars show the influence with only that variable.
(TIF)

Figure S12 Maxent Receiver Operator Characteristic (ROC) curves and Area Under the Curve (AUC) values for training and test data for the auk seasonal models.
(TIF)

Figure S13 Spatial distribution of strandings of marine turtle (A) and cetacean (B) families in spring (A), summer (B), autumn (C), and winter (D).
(TIF)

Table S1 Number of training (and test) samples used in seasonal maximum entropy models.
(DOCX)

Table S2 List of species observed in the study area, sources (see footnotes), and protection statuses.
(DOCX)
