# Supplementary figures and images for: Understanding the Distribution of Marine Megafauna in the English Channel Region: Identifying Key Habitats for Conservation within the Busiest Seaway on Earth
Source: PLoS One. 2014 Feb 28;9(2):e89720. doi: 10.1371/journal.pone.0089720 (PMC3938532; doi:10.1371/journal.pone.0089720)

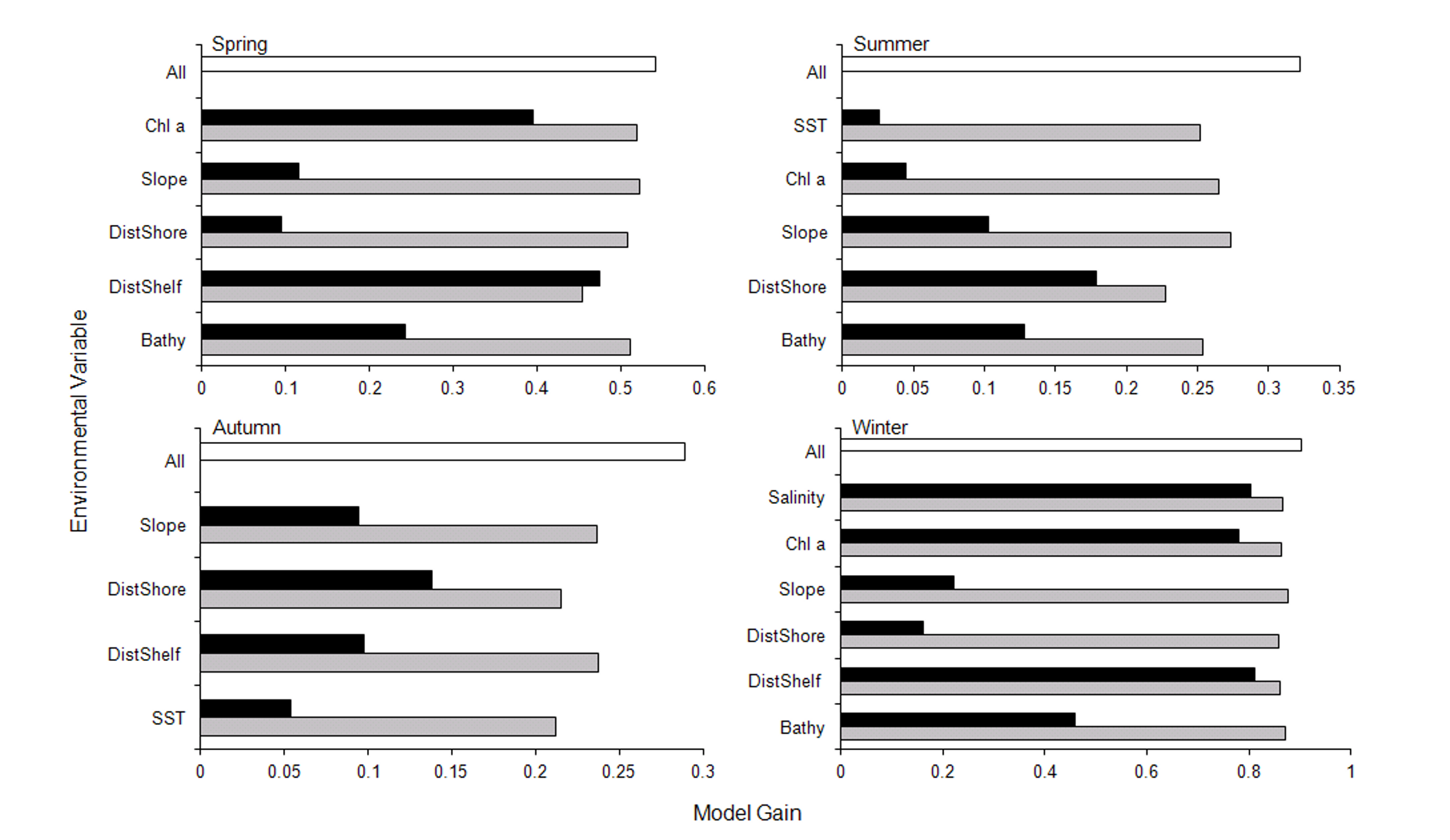

Supplement: Figure S1 — Maxent results of jackknife analyses of the environmental variable importance for harbour porpoise predictions. Grey bars show the performance (in terms of training gain) of the global model without each variable and black bars show the influence with only that variable. (TIF) [file pone.0089720.s001.tif]

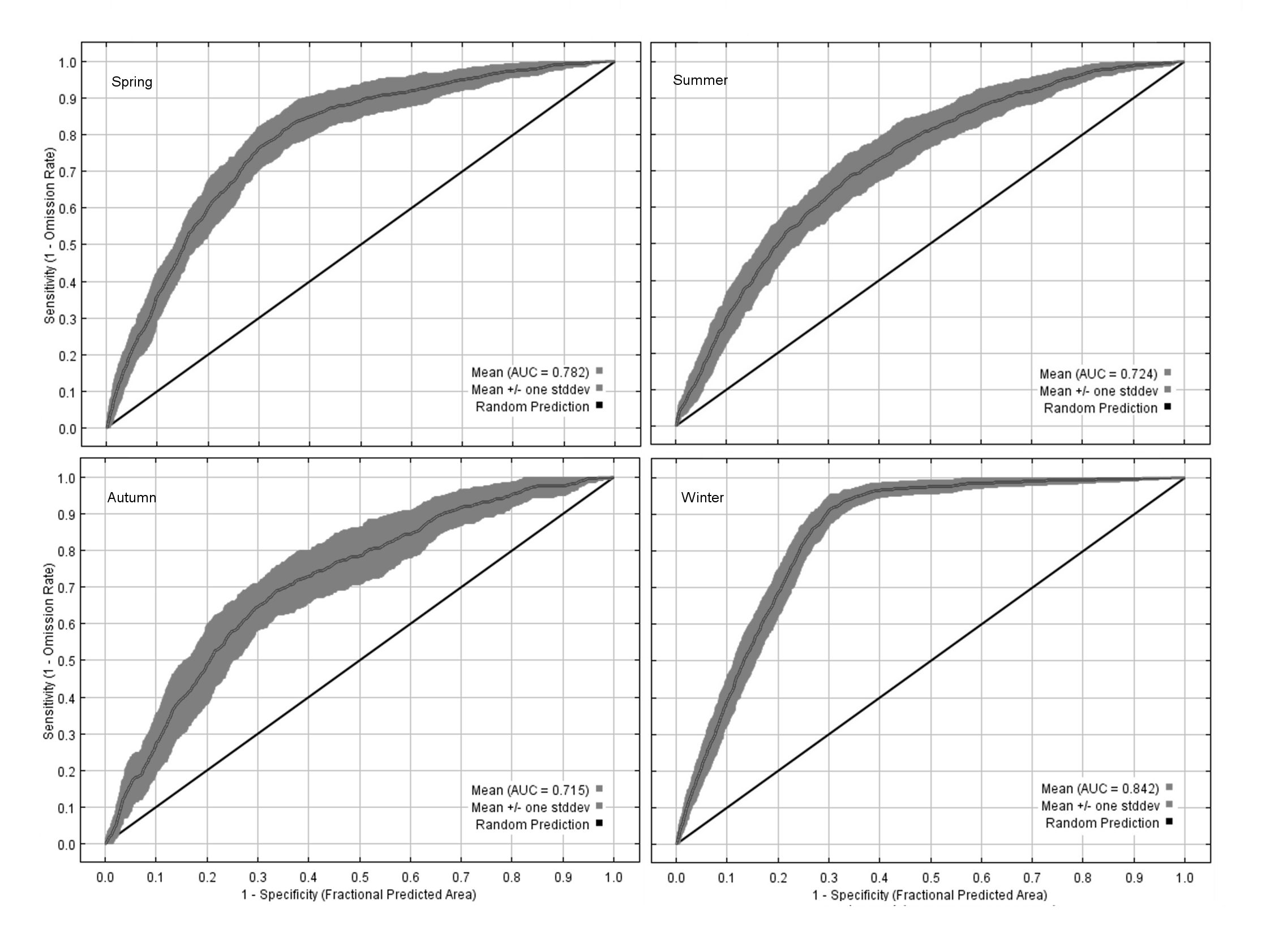

Supplement: Figure S2 — Maxent Receiver Operator Characteristic (ROC) curves and Area Under the Curve (AUC) values for training and test data for the harbour porpoise seasonal models. (TIF) [file pone.0089720.s002.tif]

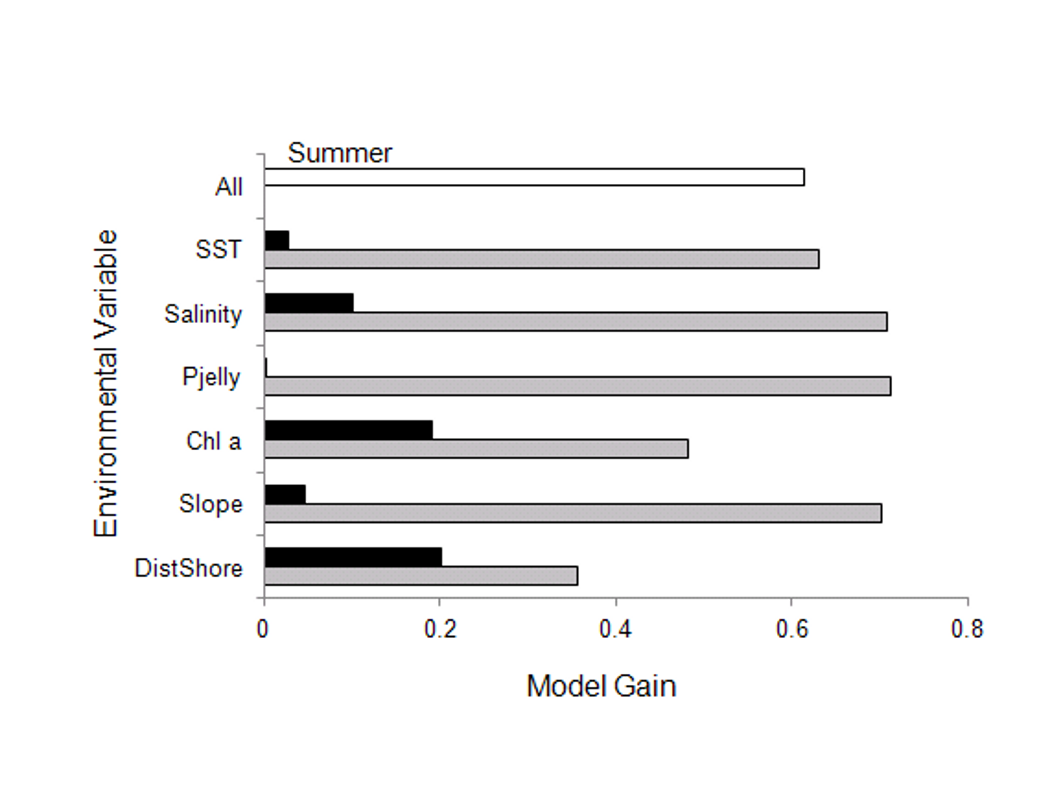

Supplement: Figure S3 — Maxent results of jackknife analyses of the environmental variable importance for the leatherback prediction. Grey bars show the performance (in terms of training gain) of the global model without each variable and black bars show the influence with only that variable. (TIF) [file pone.0089720.s003.tif]

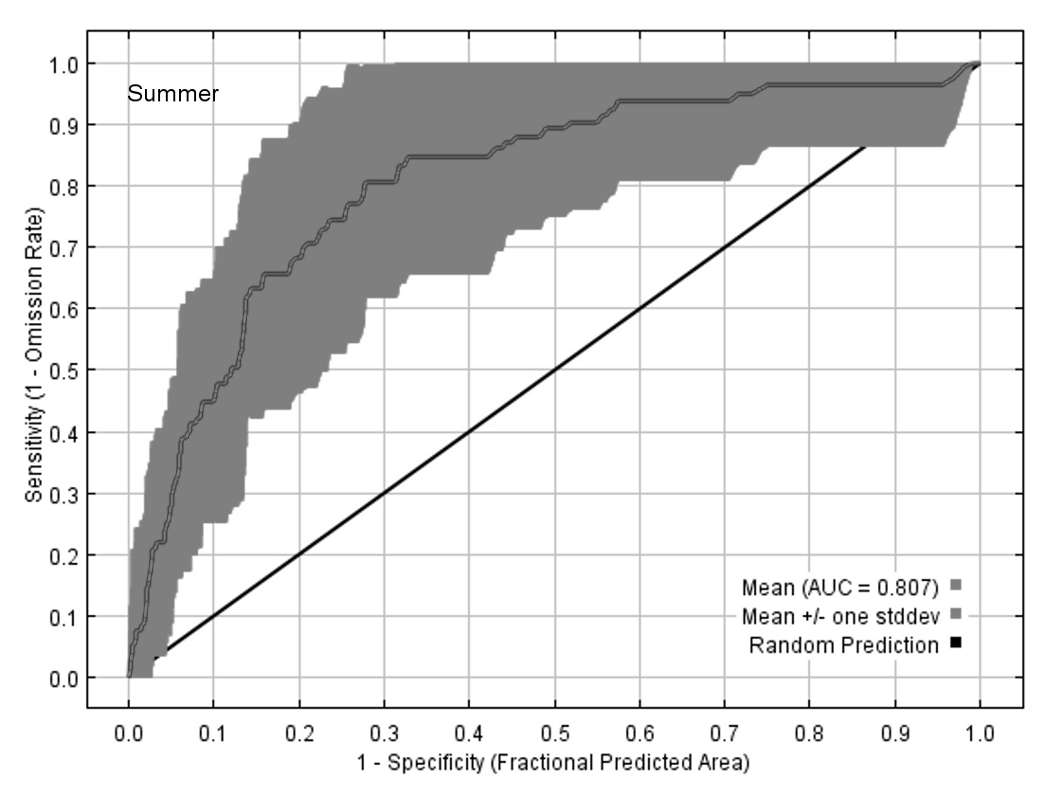

Supplement: Figure S4 — Maxent Receiver Operator Characteristic (ROC) curve and Area Under the Curve (AUC) value for training and test data for the leatherback turtle model. (TIF) [file pone.0089720.s004.tif]

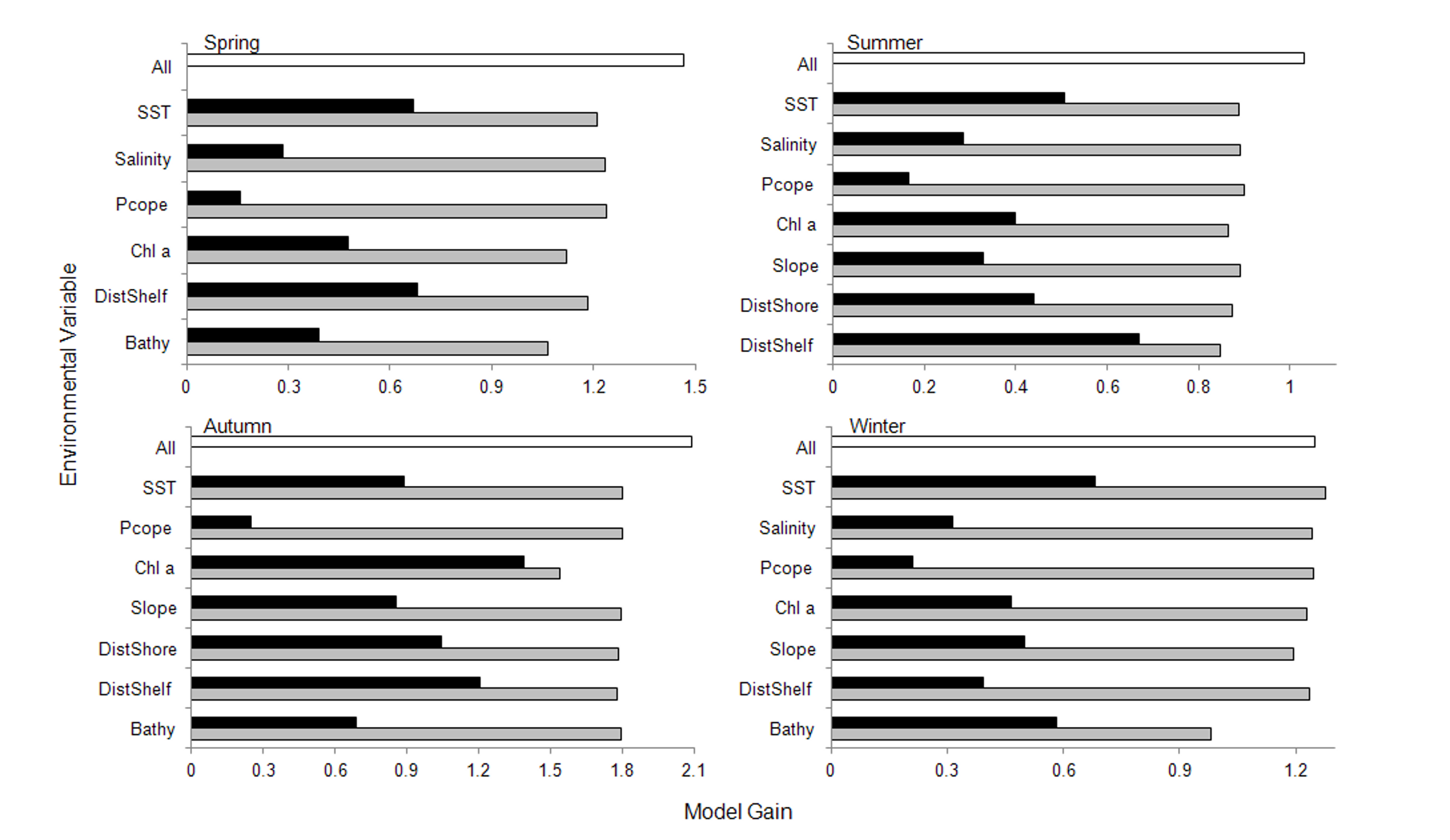

Supplement: Figure S5 — Maxent results of jackknife analyses of the environmental variable importance for basking shark predictions. Grey bars show the performance (in terms of training gain) of the global model without each variable and black bars show the influence with only that variable. (TIF) [file pone.0089720.s005.tif]

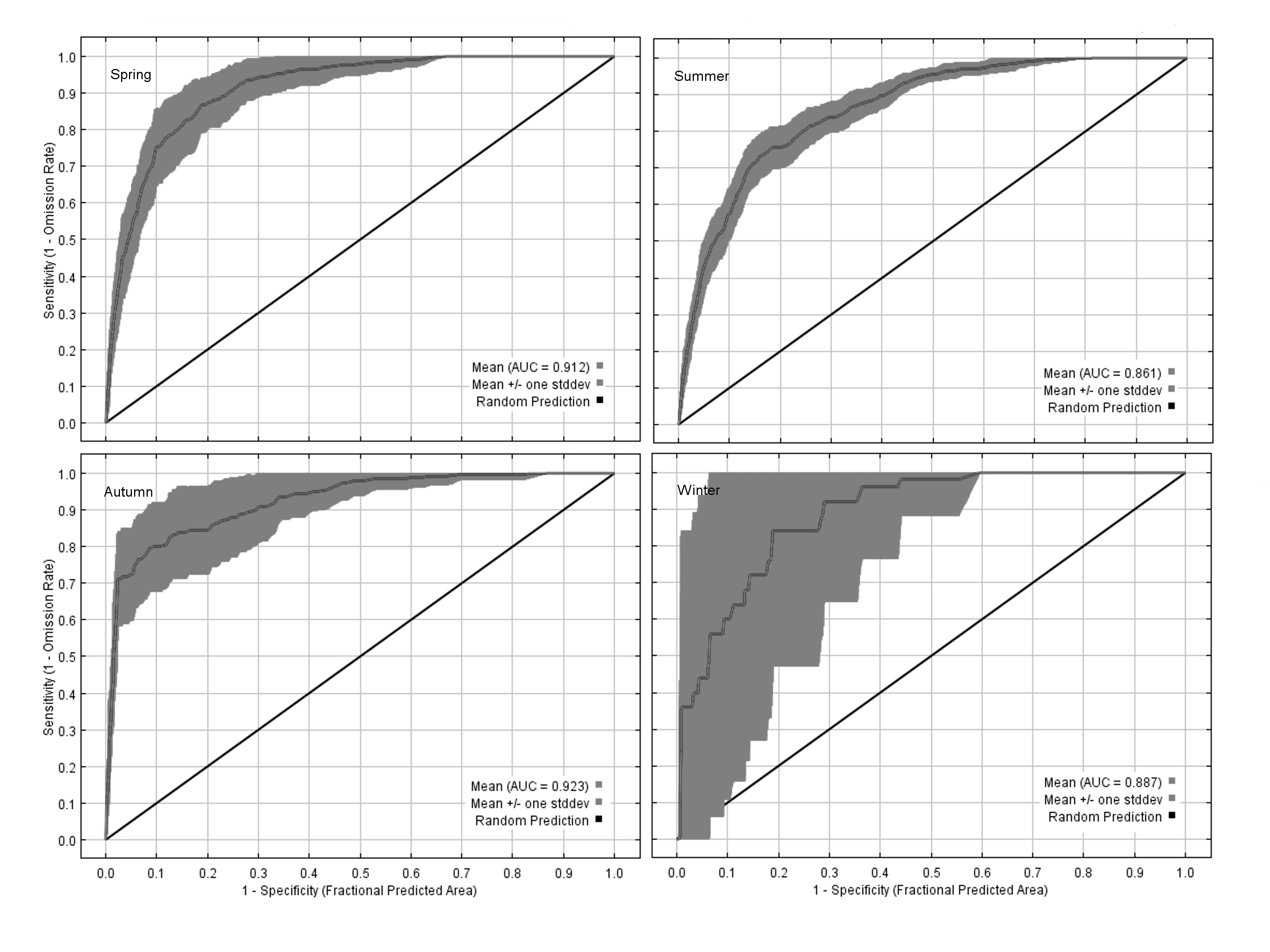

Supplement: Figure S6 — Maxent Receiver Operator Characteristic (ROC) curves and Area Under the Curve (AUC) values for training and test data for the basking shark seasonal models. (TIF) [file pone.0089720.s006.tif]

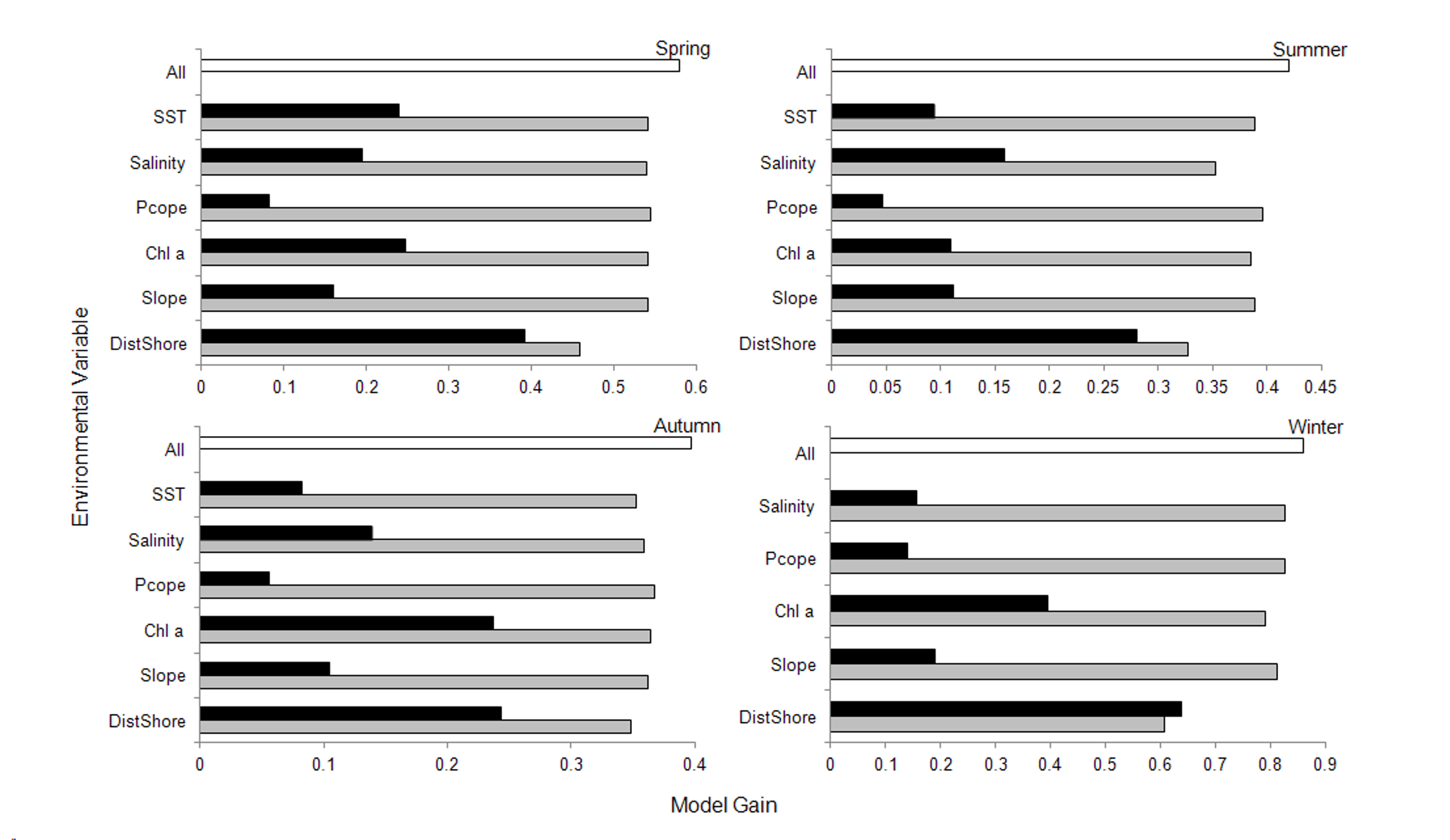

Supplement: Figure S7 — Maxent results of jackknife analyses of the environmental variable importance for gannet predictions. Grey bars show the performance (in terms of training gain) of the global model without each variable and black bars show the influence with only that variable. (TIF) [file pone.0089720.s007.tif]

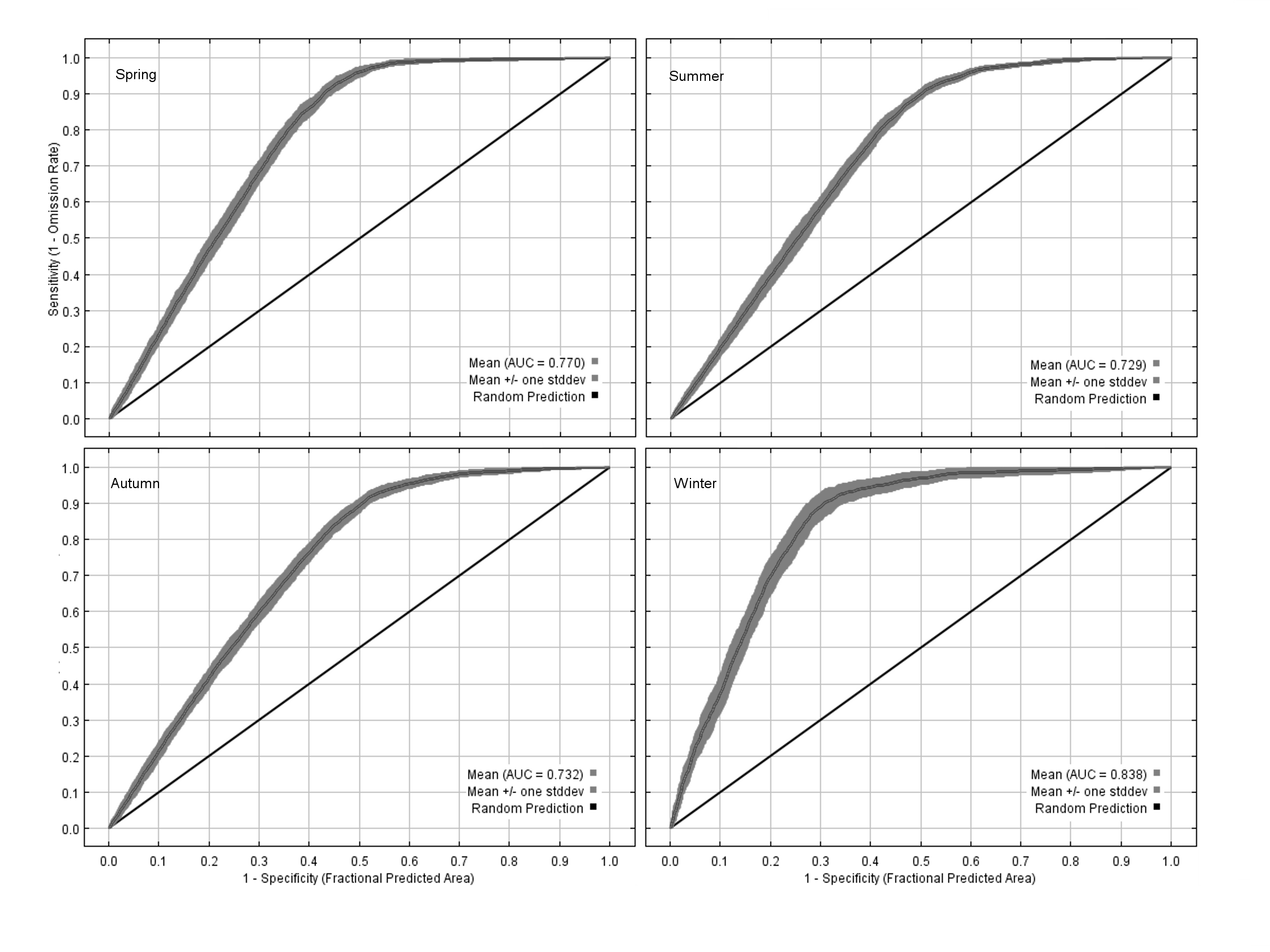

Supplement: Figure S8 — Maxent Receiver Operator Characteristic (ROC) curves and Area Under the Curve (AUC) values for training and test data for the gannet seasonal models. (TIF) [file pone.0089720.s008.tif]

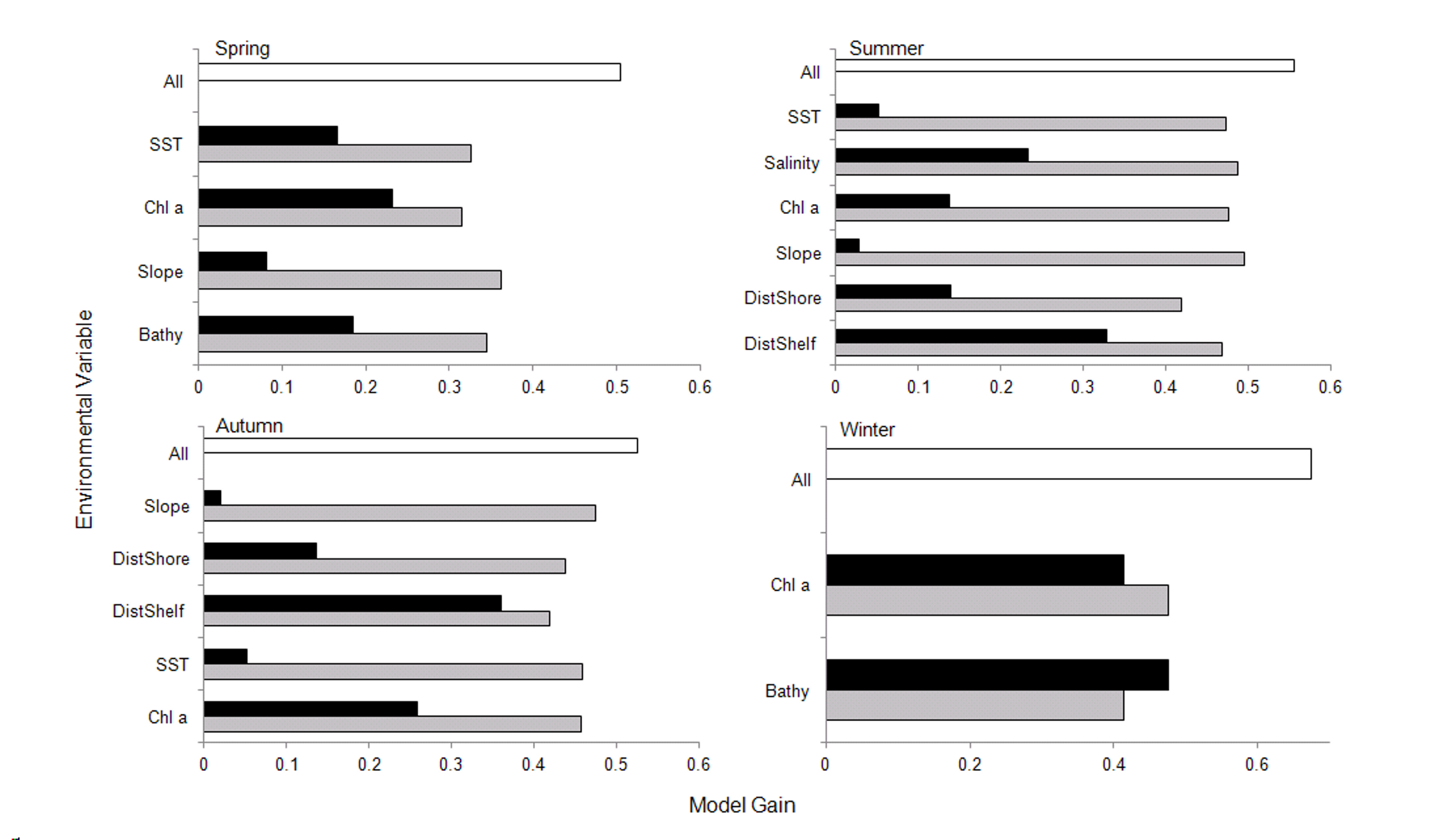

Supplement: Figure S9 — Maxent results of jackknife analyses of the environmental variable importance for dolphin predictions. Grey bars show the performance (in terms of training gain) of the global model without each variable and black bars show the influence with only that variable. (TIF) [file pone.0089720.s009.tif]

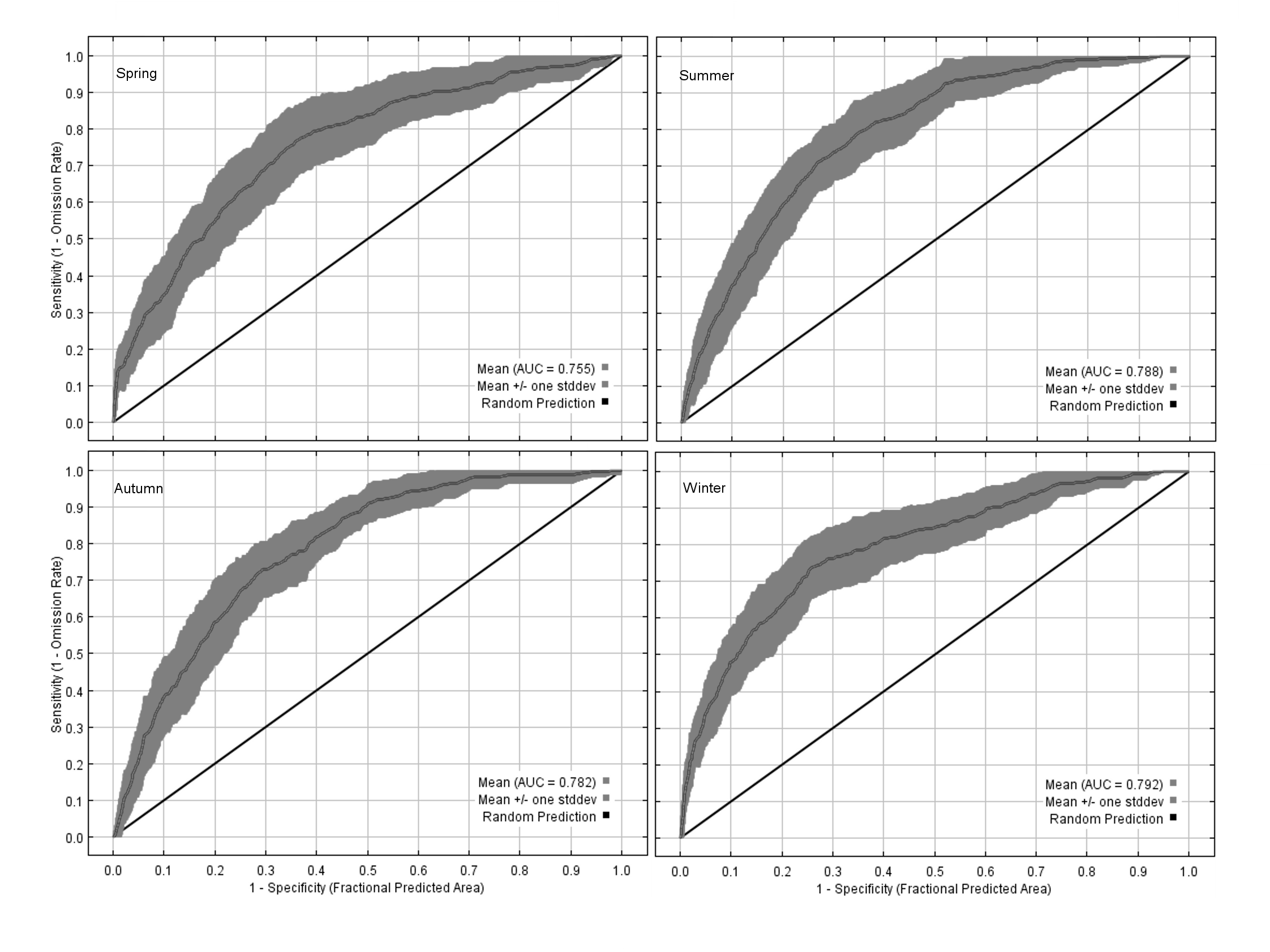

Supplement: Figure S10 — Maxent Receiver Operator Characteristic (ROC) curves and Area Under the Curve (AUC) values for training and test data for the dolphin seasonal models. (TIF) [file pone.0089720.s010.tif]

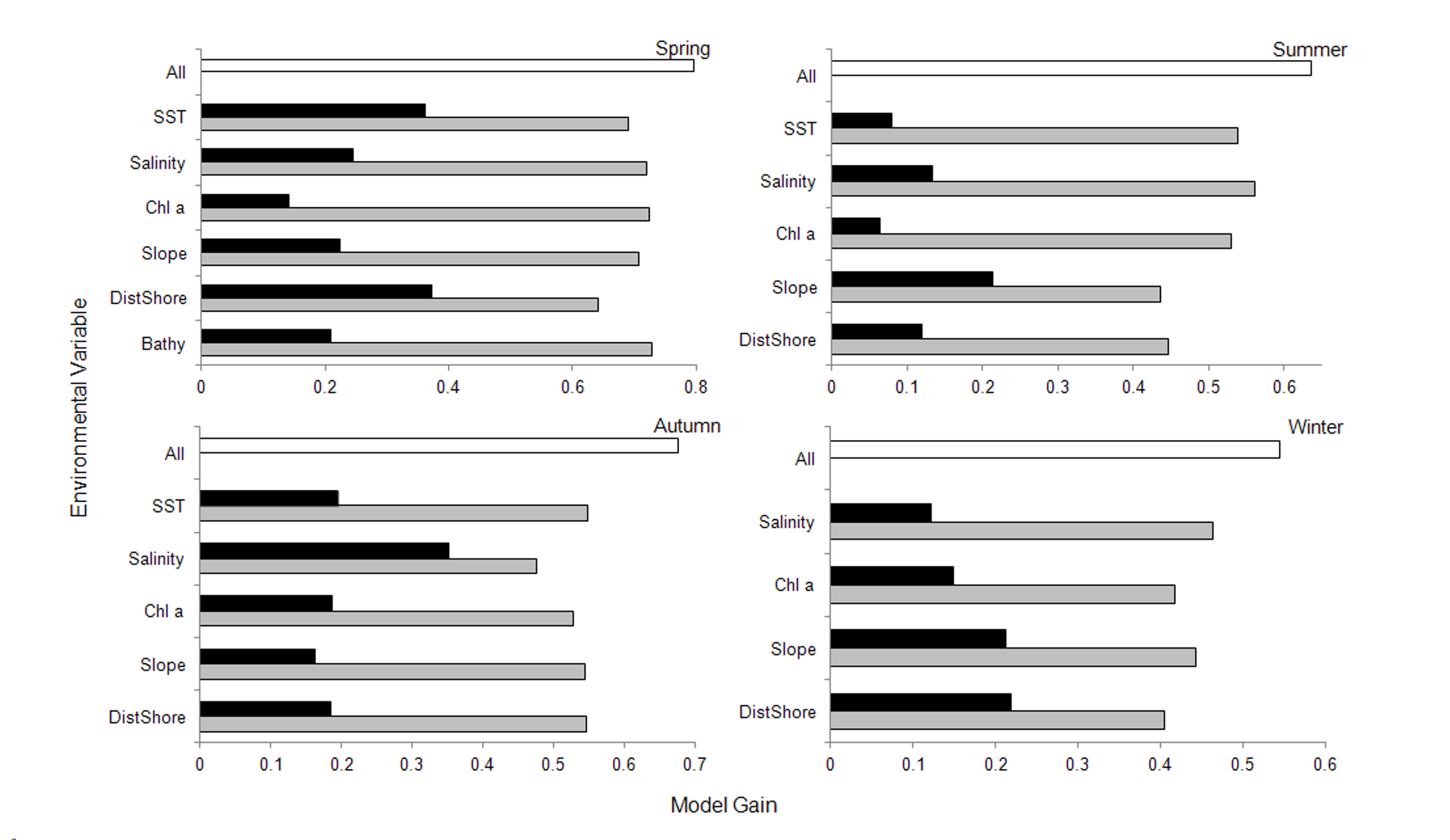

Supplement: Figure S11 — Maxent results of jackknife analyses of the environmental variable importance for auk predictions. Grey bars show the performance (in terms of training gain) of the global model without each variable and black bars show the influence with only that variable. (TIF) [file pone.0089720.s011.tif]

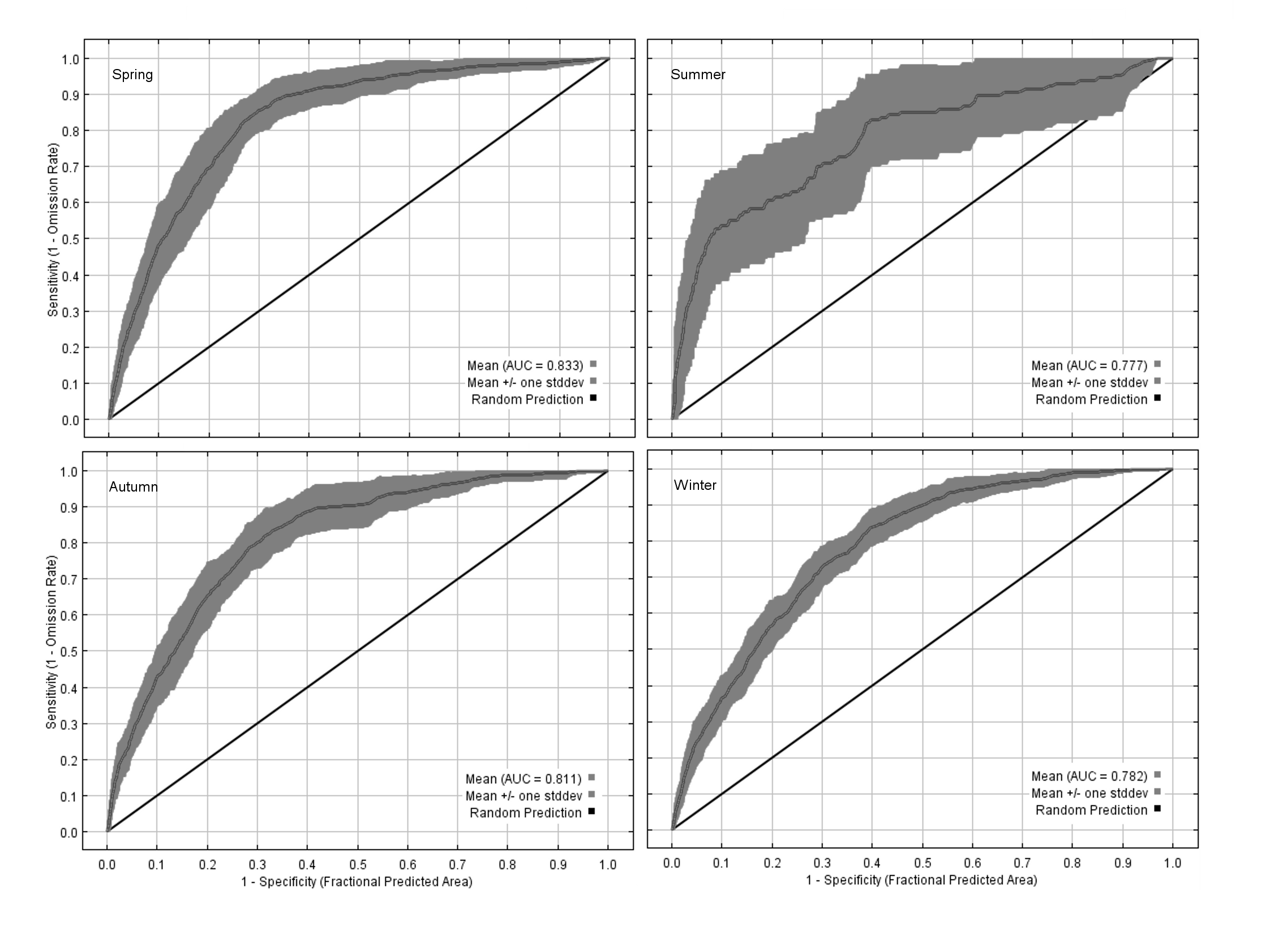

Supplement: Figure S12 — Maxent Receiver Operator Characteristic (ROC) curves and Area Under the Curve (AUC) values for training and test data for the auk seasonal models. (TIF) [file pone.0089720.s012.tif]

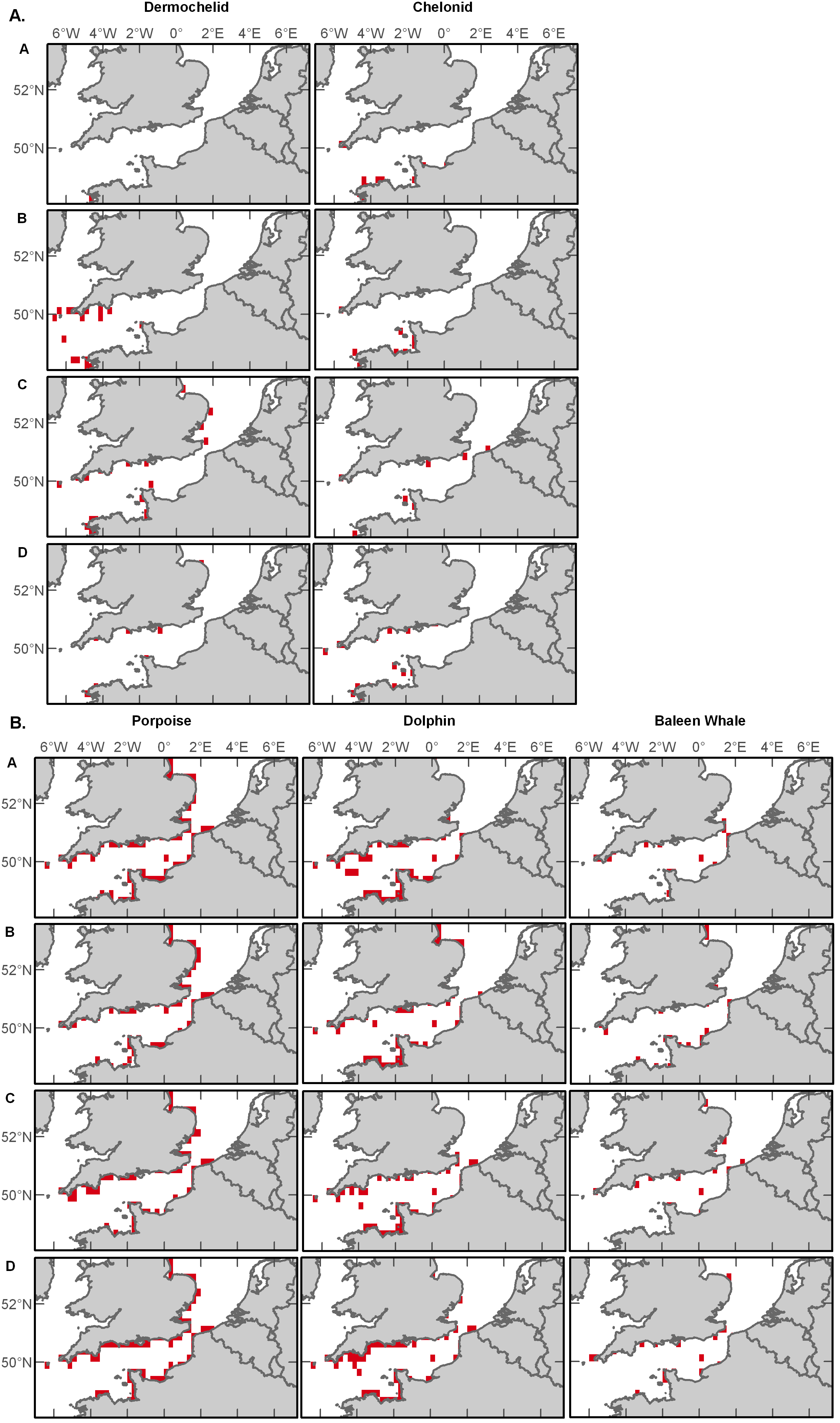

Supplement: Figure S13 — Spatial distribution of strandings of marine turtle (A) and cetacean (B) families in spring (A), summer (B), autumn (C), and winter (D). (TIF) [file pone.0089720.s013.tif]
