# Supplementary material for: Understanding the Distribution of Marine Megafauna in the English Channel Region: Identifying Key Habitats for Conservation within the Busiest Seaway on Earth
Source: PLoS One. 2014 Feb 28;9(2):e89720. doi: 10.1371/journal.pone.0089720 (PMC3938532; doi:10.1371/journal.pone.0089720)
Supplement: Table S1 — Number of training (and test) samples used in seasonal maximum entropy models. (DOCX) [file pone.0089720.s014.docx]

Table S1. Number of training (and test) samples used in seasonal maximum entropy models.

| Model | Spring  MAM | Summer  JJA | Autumn  SON | Winter  DJF |
| --- | --- | --- | --- | --- |
| Harbour porpoise | 1,266  (53) | 1,249  (54) | 173  (7) | 1,536  (64) |
| Leatherback turtle | – | 78  (3) | – | – |
| Basking shark | 551  (23) | 1,179  (49) | 314  (13) | 25  (2) |
| Gannet | 5,360  (223) | 7,541  (314) | 6,052  (252) | 2,234  (93) |
| Dolphin | 500  (21) | 544  (23) | 444  (18) | 498  (21) |
| Auk | 932  (39) | 122  (5) | 735  (31) | 1,118  (47) |
