# Supplementary material for: Understanding the Distribution of Marine Megafauna in the English Channel Region: Identifying Key Habitats for Conservation within the Busiest Seaway on Earth
Source: PLoS One. 2014 Feb 28;9(2):e89720. doi: 10.1371/journal.pone.0089720 (PMC3938532; doi:10.1371/journal.pone.0089720)
Supplement: Table S2 — List of species observed in the study area, sources (see footnotes), and protection statuses. (DOCX) [file pone.0089720.s015.docx]

Table S2. List of species observed in the study area, sources (see footnotes), and protection statuses.

| Umbrella Group | Common Name | Scientific Name | Sighting/  Stranding | Data Source | IUCN Status | EU Protection |
| --- | --- | --- | --- | --- | --- | --- |
| Cetacean | Minke Whale | *Balaenoptera acutorostrata* | SI, ST | 1,2,4,7,10,12,13,15 | Least Concern | UKBS, EUHD |
| Cetacean | Sei Whale | *Balaenoptera borealis* | ST | 4,10 | Endangered | UKBS, EUHD |
| Cetacean | Fin Whale | *Balaenoptera physalus* | SI, ST | 2,4,10,15 | Endangered | UKBS, EUHD |
| Cetacean | Short-beaked Common Dolphin | *Delphinus delphis* | SI, ST | 1,2,4,6,7,10,12,13,15 | Least Concern | UKBS, EUHD |
| Cetacean | Long-finned Pilot Whale | *Globicephala melas* | SI, ST | 1,2,4,10,13,15 | Data Deficient | UKBS, EUHD |
| Cetacean | Risso's Dolphin | *Grampus griseus* | SI, ST | 1,2,4,7,10,13,15 | Least Concern | UKBS, EUHD |
| Cetacean | Northern Bottlenose Whale | *Hyperoodon ampullatus* | SI, ST | 2,4,10 | Data Deficient | UKBS, EUHD |
| Cetacean | Pygmy Sperm Whale | *Kogia breviceps* | ST | 4 | Data Deficient | EUHD |
| Cetacean | Fraser's Dolphin | *Lagenodelphis hosei* | ST | 10 | Least Concern | – |
| Cetacean | Atlantic White-sided Dolphin | *Lagenorhynchus acutus* | SI, ST | 2,4,10,15 | Least Concern | UKBS, EUHD |
| Cetacean | White-beaked Dolphin | *Lagenorhynchus albirostris* | SI, ST | 1,2,4,6,9,10,15 | Least Concern | UKBS, EUHD |
| Cetacean | Humpback Whale | *Megaptera novaeangliae* | SI, ST | 2,4,10,15 | Least Concern | UKBS, EUHD |
| Cetacean | Sowerby's Beaked Whale | *Mesoplodon bidens* | ST | 4,10 | Data Deficient | UKBS, EUHD |
| Cetacean | Killer Whale | *Orcinus orca* | SI, ST | 2,4,13,15 | Data Deficient | UKBS, EUHD |
| Cetacean | Harbour Porpoise | *Phocoena phocoena* | SI, ST | 1,2,4,6,7,9^a^,10,12,13,15 | Least Concern | OSPAR, UKBS, EUHD |
| Cetacean | Sperm Whale | *Physeter macrocephalus* | SI, ST | 2,4,10 | Vulnerable | UKBS, EUHD |
| Cetacean | False Killer Whale | *Pseudorca crassidens* | SI | 15 | Data Deficient | EUHD |
| Cetacean | Striped Dolphin | *Stenella coeruleoalba* | SI, ST | 2,4,10,15 | Least Concern | UKBS, EUHD |
| Cetacean | Bottlenose Dolphin | *Tursiops truncatus* | SI, ST | 1,2,4,6,7,10,12,13,15 | Least Concern | UKBS, EUHD |
| Cetacean | Cuvier's Beaked Whale | *Ziphius cavirostris* | SI, ST | 4,10,15 | Least Concern | UKBS, EUHD |
| Large Pelagic Fish | Thresher Shark spp. | *Alopias spp.* | SI | 2 | Vulnerable | – |
| Large Pelagic Fish | Basking Shark | *Cetorhinus maximus* | SI | 2,5,6,11,13,15 | Vulnerable | OSPAR, UKBS |
| Large Pelagic Fish | Porbeagle Shark | *Lamna nasus* | SI | 2 | Vulnerable | OSPAR, UKBS |
| Large Pelagic Fish | Ocean Sunfish | *Mola mola* | SI | 2,5,6,12,13 | Not Evaluated | – |
| Large Pelagic Fish | Blue Shark | *Prionace glauca* | SI | 2 | Near Threatened | – |
| Marine Turtle | Loggerhead Sea Turtle | *Caretta caretta* | SI, ST | 3,8 | Endangered | OSPAR, UKBS, EUHD |
| Marine Turtle | Green Sea Turtle | *Chelonia mydas* | ST | 3,8 | Endangered | EUHD |
| Marine Turtle | Leatherback Sea Turtle | *Dermochelys coriacea* | SI, ST | 2,3,8 | Critically Endangered | OSPAR, UKBS, EUHD |
| Marine Turtle | Kemp's ridley Sea Turtle | *Lepidochelys kempii* | ST | 3,8 | Critically Endangered | EUHD |
| Pinniped | Hooded Seal | *Cystophora cristata* | ST | 10 | Vulnerable | – |
| Pinniped | Bearded Seal | *Erignathus barbatus* | ST | 10 | Least Concern | – |
| Pinniped | Grey Seal | *Halichoerus grypus* | SI, ST | 2,6,7,10,13,14 | Least Concern | EUHD |
| Pinniped | Harp Seal | *Pagophilus groenlandicus* | ST | 10 | Least Concern | – |
| Pinniped | Harbour Seal | *Phoca vitulina* | SI, ST | 2,6,7,10 | Least Concern | UKBS, EUHD |
| Pinniped | Ringed Seal | *Pusa hispida* | ST | 10 | Least Concern | EUHD |
| Seabird | Razorbill | *Alca torda* | SI | 2,16^a^ | Least Concern | – |
| Seabird | Cory's Shearwater | *Calonectris diomedea* | SI | 2,16^a^ | Least Concern | EUBD |
| Seabird | Black Tern | *Chlidonias nigra* | SI | 2,16^a^ | Not Evaluated | – |
| Seabird | Black-Headed Gull | *Chroicocephalus ridibundus* | SI | 2,16^a^ | Least Concern | EUBD |
| Seabird | Atlantic Puffin | *Fratercula arctica* | SI | 2,16^a^ | Least Concern | – |
| Seabird | Northern Fulmar | *Fulmaris glacialis* | SI | 2,16^a^ | Not Evaluated | – |
| Seabird | Black-throated Diver | *Gavia arctica* | SI | 2,16^a^ | Least Concern | UKBS, EUBD |
| Seabird | Great Northern Diver | *Gavia immer* | SI | 2,16^a^ | Least Concern | EUBD |
| Seabird | Red-throated Diver | *Gavia stellata* | SI | 2,16^a^ | Least Concern | EUBD |
| Seabird | European Storm-petrel | *Hydrobates pelagicus* | SI | 2,16^a^ | Least Concern | EUBD |
| Seabird | Herring Gull | *Larus argentatus* | SI | 2,16^a^ | Least Concern | UKBS, EUBD |
| Seabird | Yellow-Legged Gull | *Larus cachinnans* | SI | 2 | Least Concern | EUBD |
| Seabird | Common Gull | *Larus canus* | SI | 2,16^a^ | Least Concern | EUBD |
| Seabird | Lesser Black-Backed Gull | *Larus fuscus* | SI | 2,16^a^ | Least Concern | OSPAR, EUBD |
| Seabird | Greater Black-Backed Gull | *Larus marinus* | SI | 2,16^a^ | Least Concern | EUBD |
| Seabird | Mediterranean Gull | *Larus melanocephalus* | SI | 2,16^a^ | Least Concern | – |
| Seabird | Little Gull | *Larus minutus* | SI | 2,16^a^ | Least Concern | – |
|  |  |  |  |  |  |  |
| Seabird | Velvet Scoter | *Melanitta fusca* | SI | 2,16^a^ | Endangered | EUBD |
| Seabird | Common Scoter | *Melanitta nigra* | SI | 2,16^a^ | Least Concern | UKBS, EUBD |
| Seabird | Gannet | *Morus bassanus* | SI | 2,16^a^ | Least Concern | – |
| Seabird | Wilson's Storm-petrel | *Oceanites oceanicus* | SI | 2 | Least Concern | – |
| Seabird | Leach's Petrel | *Oceanodroma leucorhoa* | SI | 2,16^a^ | Least Concern | EUBD |
| Seabird | European Shag | *Phalacrocorax aristotelis* | SI | 2,16^a^ | Least Concern | EUBD |
| Seabird | Great Cormorant | *Phalacrocorax carbo carbo* | SI | 2,16^a^ | Not Evaluated | – |
| Seabird | Grey Phalarope | *Phalaropus fulicarius* | SI | 2,16^a^ | Least Concern | – |
| Seabird | Great Crested Grebe | *Podiceps cristatus* | SI | 2,16^a^ | Least Concern | – |
| Seabird | Fea's Petrel | *Pterodroma feae* | SI | 2 | Near Threatened | EUBD |
| Seabird | Little Shearwater | *Puffinus assimilis* | SI | 2 | Least Concern | OSPAR, EUBD |
| Seabird | Great Shearwater | *Puffinus gravis* | SI | 2,16^a^ | Least Concern | – |
| Seabird | Sooty Shearwater | *Puffinus griseus* | SI | 2,16^a^ | Near Threatened | – |
| Seabird | Balearic Shearwater | *Puffinus mauretanicus* | SI | 2,16^a^ | Critically Endangered | OSPAR, UKBS, EUBD |
| Seabird | Manx Shearwater | *Puffinus puffinus* | SI | 2,16^a^ | Least Concern | – |
| Seabird | Black-legged Kittiwake | *Rissa tridactyla* | SI | 2,16^a^ | Least Concern | – |
| Seabird | Long-Tailed Skua | *Stercorarius longicaudus* | SI | 2,16^a^ | Least Concern | – |
| Seabird | Arctic Skua | *Stercorarius parasiticus* | SI | 2,16^a^ | Least Concern | UKBS |
| Seabird | Pomarine Skua | *Stercorarius pomarinus* | SI | 2,16^a^ | Least Concern | – |
| Seabird | Great Skua | *Stercorarius skua* | SI | 2,16^a^ | Least Concern | – |
| Seabird | Roseate Tern | *Sterna dougallii* | SI | 2,16^a^ | Least Concern | OSPAR, UKBS |
| Seabird | Common Tern | *Sterna hirundo* | SI | 2,16^a^ | Least Concern | – |
| Seabird | Arctic Tern | *Sterna paradisaea* | SI | 2,16^a^ | Least Concern | – |
| Seabird | Sandwich Tern | *Thalasseus sandvicensis* | SI | 2,16^a^ | Not Evaluated | – |
| Seabird | Common Guillemot | *Uria aalge* | SI | 2,16^a^ | Least Concern | EUBD |
| Seabird | Sabine's Gull | *Xema sabini* | SI | 2,16^a^ | Least Concern | – |

1. Joint Cetacean Database; 2. Marinelife; 3. Marine Environmental Monitoring; 4. Cetacean Strandings Investigation Programme; 5. Marine Conservation Society; 6. Wildfowl and Wetlands Trust Consulting; 7. Sea Mammal Research Unit; 8. Aquarium La Rochelle; 9. Marine Conservation Research International and International Fund for Animal Welfare; 10. Observatoire PELAGIS; 11. Association Pour l'Etude et la Conservation des Sélaciens; 12. Iroise Park Naturel Marin; 13. University of Exeter; 14. Cornwall Seal Group; 15. Cornwall Wildlife Trust Seaquest Southwest; 16. European Seabirds at Sea.

^a^Datasets that were accessed in part or in whole through the OBIS-SEAMAP porthole [Read AJ, Halpin PN, Crowder LB, Best BD, Fujioka E (2009) OBIS-SEAMAP: mapping marine mammals, birds and turtles. Available: http://seamap.env.duke.edu, Accessed 15 September 2009.].
